# Using technology to prevent fraud in high stakes national school examinations: Evidence from Indonesia^☆^^☆☆^

**DOI:** 10.1016/j.jdeveco.2024.103307

**Published:** 2024-09

**Authors:** Emilie Berkhout, Menno Pradhan, Rahmawati, Daniel Suryadarma, Arya Swarnata

**Affiliations:** a Youth Impact, Botswana; b Vrije Universiteit Amsterdam, University of Amsterdam, Tinbergen Institute, AIGHD, Netherlands; c Government of Indonesia, Indonesia; d Asian Development Bank Institute, Japan; e The SMERU Research Institute, Indonesia

**Keywords:** Education, National examinations, Cheating, Education technology, Indonesia

## Abstract

Cheating reduces the signaling value of examinations. It also shifts the focus of teachers and students away from learning. Combating widespread cheating is difficult as students, teachers, and bureaucrats all benefit from high reported grades. We evaluate the impact of computer-based testing (CBT), an at-scale policy implemented by the Indonesian government to reduce widespread cheating in the national examinations. Exploiting the phased roll-out of the program from 2015 to 2019, we find that test scores declined dramatically, by 0.5 standard deviations, after the introduction of CBT. Schools with response patterns that indicated cheating prior to CBT adoption experienced a steeper decline. The effect is similar between schools with and without access to a computer lab, indicating that the reduction in the opportunity to cheat is the main reason for the test score decline. In districts with high adoption of CBT, schools that still used paper-based exams cheated less and scored lower, indicating spillovers of CBT. The results highlight the potential role of technology in improving the effectiveness in efforts to overcome collusive behavior in the education sector.

## Highlights

• Widespread cheating in national school examinations is hard to combat.
• The Indonesian government implemented computer-based testing to eradicate cheating.
• The policy was technology-based and implemented at scale.
• Our double difference estimates show a dramatic decline in test scores.
• The decline was caused mainly by the reduction in the opportunity to cheat.

## 1 Introduction

Cheating happens in high stakes school examinations, from the ‘cheating mafia’ in India (Anderman, 2015) to fraudulent practices in prestigious high schools in the United States (Safi, 2018). Systemic cheating is difficult to eliminate, as all stakeholders benefit. Students and teachers need to exert less effort to attain higher grades compared to the situation where cheating is not feasible. Since official grades are what teachers and bureaucrats are held accountable for, they may prefer to allow cheating. Honest test takers lose, because their results reflect poorly relative to the cheaters. When honest test takers are the minority, the costs to report cheating practices are high and the chance that the authorities seriously attempt to reduce cheating is low (Borcan et al., 2017). Therefore, the honest students and teachers may be compelled to cheat too. The resulting equilibrium is characterized by sustained cheating practices, which in turn reduces the signaling value of examinations.

We evaluate the Indonesian government’s flagship policy to eliminate cheating in school national examinations: computer-based testing (CBT). Cheating in paper-based national exams in Indonesia was widespread. It has been reported in the popular press (e.g. Economist, 2011, Sundaryani, 2015, Jong, 2015). Reported cheating ranged from students copying each other’s answers to teachers and principals providing answer keys to students prior to or on the exam day. Anecdotes of teachers correcting students’ answers before grading also exist.

In 2015, the extent of cheating became apparent when the central government began measuring a school “integrity index” (Rahmawati and Asrijanty, 2016). The integrity index identifies cheating through suspicious answer patterns, a method that has been validated in schools in Chicago (Jacob and Levitt, 2003) and in India (Singh, 2022), and has been used in multiple studies to measure cheating in exams (Battistin et al., 2017, Martinelli et al., 2018). In Italy, a similar method was used to sanction schools for cheating on a standardized national test in primary and high schools (Lucifora and Tonello, 2020). Classrooms with identical answer strings or counter-intuitive performance, such as scoring high on difficult items while incorrectly answering easier items, are given a lower integrity index. The results revealed widespread cheating. One-third of the schools were flagged by the Ministry of Education as suspicious, compared to 5 percent of the classrooms in Chicago (Jacob and Levitt, 2003) and 5 percent in Italy (Angrist et al., 2017). In Mexico, 7 percent of high school exams were flagged as suspicious (Martinelli et al., 2018), which increased to 32 percent after two years of monetary incentives based on test scores for students and teachers.

The Ministry of Education (MoE) introduced CBT in 2015 with the explicit and singular aim to eliminate cheating (Rahmawati and Asrijanty, 2016). With CBT, the test items are drawn directly from a server, so test versions vary across students even in the same examination room. Cheating is virtually impossible as teachers and students do not know the questions beforehand, and thousands of test versions are available. The different test items also void the students’ ability to work together during the exam. In addition, teachers cannot change students’ answers, because the computer program grades the exam.

We implement a difference-in-difference analysis for each cohort of schools that switched to CBT between 2017 and 2019 as the program was rolled out across the country. The program started with 40 junior secondary schools in 2015. By 2019, 78 percent of Indonesia’s junior secondary schools (43,841 schools with 3,554,556 exam takers) participated in CBT. We use publicly available data at the school level on the average exam score, the variance, the number of students taking the national exam and the integrity index. We use the Callaway–Sant’Anna approach to exploit the staggered implementation for measuring the program’s impacts (Callaway and Sant’Anna, 2021), taking heterogeneous treatment effects across cohorts and treatment anticipation into account.

We find that school level exam scores decreased on average by 6.3 points on a 1–100 scale (0.5 standard deviations) in the first year of participation in CBT. We also find that the negative impact on the mathematics score, a subject that students find difficult and thus are more likely to cheat on, was larger than for the Indonesian or English, which are considered easier subjects. To confirm that this effect was due to a reduction in cheating rather than a change in the test-taking mode, we split the sample by high and low integrity (defining low integrity as being flagged by the Ministry) and availability of a computer lab in 2015 (used as a proxy for familiarity with working on computers). We find that the impact estimates are much more affected by the integrity score than by whether or not the school had computers, indicating that the drop in test score was indeed because of reduced cheating.

We also find that the spread of test scores within schools increased by 0.6 standard deviations as a result of CBT, which, when compared to a within-school standard deviation of about 5.5 in comparison schools, suggests that CBT was better able to distinguish between high and low-performing students. Finally, we show that post CBT adoption, the correlations between exam scores and district characteristics are more in line with expectations based on the literature. These findings indicate that the exam results under CBT provide a better signal of true learning levels.

In districts where CBT was adopted at a faster pace, the integrity index of schools that were still doing paper-based tests rose faster and their test scores declined more. The estimates indicate that the integrity index of schools still taking the paper-based test on average increased by one point (on a 1–100 scale) as the share of schools in the same district that adopted CBT expanded by 10 percentage points. These spillover effects were concentrated among schools located in districts with initially high cheating and only occurred when initially cheating schools switched to CBT. These findings suggest that the roll-out of CBT affected local cheating practices.

We use two strategies to correct for the spillover effects. We estimate our model holding the integrity index of the comparison schools constant and we allow the trend of the comparison group to vary with the share of schools that implement CBT in the district. As expected, corrected estimates are larger, but the difference between the main and the corrected coefficients is statistically insignificant.

We do not find evidence for improvements in exam scores within three years of implementation, which would point to a shift in focus from cheating to learning. With the correction for spillovers, none of the cohorts indicate that the impact of CBT diminished over time. The government abolished national exams in 2020, preventing us from estimating the effects of CBT on test scores for more than three years.

The switch to CBT is highly cost-effective. The annual cost of the national exam administration declined from about 9.2 million dollars to 2.4 million dollars, because printing and distributing the exams on paper is no longer necessary (Siddiq, 2018). Additional cost savings are also enjoyed by universities and employers, which can rely on national exams as an accurate measure of learning achievement. Although the intervention required a significant upfront investment in computers, internet, and servers, these costs are mostly fixed. Moreover, the infrastructure could support teaching and learning activities outside of exam periods.

This study makes the following contributions. First, it adds to a small literature on the effects of programs that reduce cheating in schools. These programs include cameras in classrooms in Romania (Borcan et al., 2017), random assignment of external monitors in Italy (Bertoni et al., 2013, Lucifora and Tonello, 2020), centralization of grading in New York (Dee et al., 2019), and tablet-based testing in India (Singh, 2020). All these studies find that the programs reduced cheating and, in turn, test scores. Our study is different on two aspects: the use of technology and the scale of implementation. Other nationwide efforts to reduce cheating are labor intensive. In contrast, we examine a nationwide technology-intensive effort. Although this mode of CBT is not new (for instance, see Wang et al. (2008) for CBT use in the United States), implementing CBT on a national scale for basic education in a developing country is exceptional. It required significant and continuous resource commitment and was technically complicated. Evaluating its impact provides information for other developing countries that may be interested in adopting the approach to improve the integrity and measurement of national school examinations.

Second, we contribute to a larger literature that tries to identify education interventions that could succeed at scale in a developing country setting. While the literature boasts numerous small-scale successful experiments (Angrist et al., 2020), few show evidence of success when implemented at-scale (Muralidharan and Niehaus, 2017). The successful use of technology-based interventions at scale is also relatively rare in a developing country setting (Rodriguez-Segura, 2022). Our analysis provides an example of a technology based intervention, designed and implemented at scale by the Government of Indonesia, that did succeed.

Third, we show that the introduction of CBT changed the overall cheating practices. The schools that still implemented paper-based testing cheated less and scored lower. These indirect effects are similar to the findings by Bertoni et al. (2013), who find that external monitors in one classroom also reduced cheating in other classrooms in the same school without an external monitor.

However, we find no evidence of that the effect of CBT on test scores changed over time. This is different from other contexts where the introduction of high stakes testing improves learning outcomes in contexts with little cheating (Jacob, 2005, Angrist and Lavy, 2009). Our findings also speak to the broader literature on group norms and enforcement (Feldman, 1984, Galbiati et al., 2021).

The rest of the paper proceeds as follows. In the next two sections we provide background information on the Indonesian national examination and the roll-out of CBT. Section 4 describes the data and descriptive statistics. Section 5 explains our empirical strategy. We report on the impact of CBT on exam scores in Section 6, and we conclude in the final section.

## 2 The extent of cheating in Indonesia’s national examination

The Indonesian government implements national examination at the end of junior and senior secondary school (grade 9 and 12, respectively). Students take multiple choice exams in Indonesian, English, mathematics, and science. Graduation has been independent from the national exam since 2015.^1^ However, these exams remain high-stakes. The national exam score is used to determine admission into higher education levels.^2^ This is especially true for the grade 9 exam, which we focus on in this paper. Admission into senior secondary schools is highly influenced by the grade 9 exam, as the majority of seats in senior secondary schools are allocated based on grade 9 exam scores (Berkhout et al., 2022).

High exam scores are not only important for students, but also for schools and district governments. The score contributes substantially to school and local government achievement indicators (Economist, 2011). Although there is no legislation for holding schools accountable on their exam scores, local governments consider performance on the national exam as a matter of prestige. They put pressure on school principals and teachers to achieve high grades.

As argued by Neal (2013), using the same assessment system to measure student achievement and school quality creates incentives to cheat for both the students and the educators. Anecdotal evidence indicates that cheating in national exams was indeed widespread in Indonesia (Economist, 2011, Jong, 2015). Students copied each other’s answers or used answer sheets, which they illegally bought or received from the teacher. Not only did teachers allow these cheating practices to take place, they were active participants. The exam answer sheets were collected and scanned at the provincial level and graded centrally by MoE, but the teacher could still interfere with the answer sheets beforehand, for example by correcting the wrong answers before they were sent to the provincial office.

Although the anecdotes above point to systemic cheating practices, the government only uncovered its scale in 2015. That year, the Center for Assessment and Learning (Pusmenjar or *Pusat Asesmen dan Pembelajaran*) of MoE developed an algorithm that generates a score to identify cheating at the school level. The algorithm is based on methods developed in the education literature (Hanson et al., 1987, Widiatmo, 2006, Van Der Linden and Sotaridona, 2006). The algorithm detects suspicious response patterns across students in the same schools and districts (Rahmawati and Asrijanty, 2016). It combines two cheating detection methods: (i) answer copying detection, where identical patterns of wrong and correct answers within a classroom or school are seen as an indication of answer copying and therefore increase suspicion of cheating; (ii) aberrant response detection, where unexpected patterns, for example consistently answering easier items incorrectly while getting more difficult items correctly, are seen as an indication of cheating. The second method is performed because identical wrong answers could also result from teachers incorrectly teaching the concept that the item tests. In addition, Pusmenjar adds two qualitative checks. First, Pusmenjar checks school exam results in previous years. A school that achieved uniformly correct answers and thus scored highly would be suspected of cheating if it had a track record of poor performance. Second, the school-level integrity index is validated against a qualitative measure of school quality determined by respective provincial governments and against school accreditation reports. The methods produce an index that estimates the probability that a school cheated in the exam.^3^ An integrity index, which is the complement of the probability to cheat, is then calculated for each school. The index measures cheating on a continuous scale between 0 and 100, where a lower score means that there is more evidence for cheating. Pusmenjar considers an integrity index below 70 as low integrity, between 70 and 80 as fair integrity and above 80 as high integrity.^4^

**Fig. 1:**
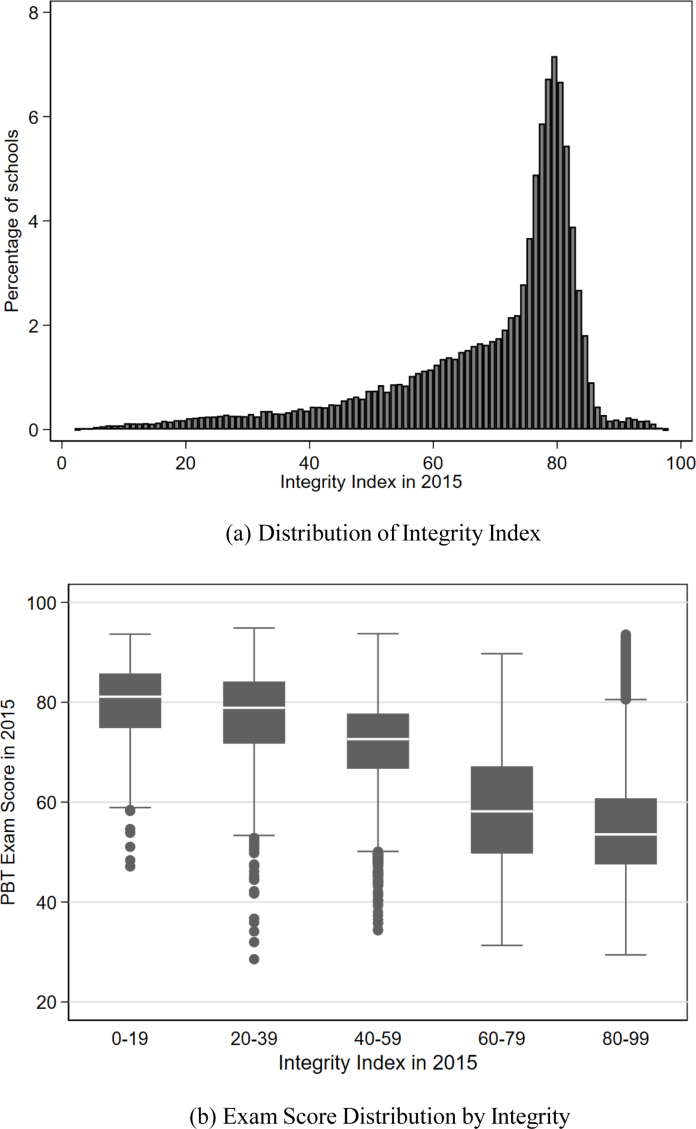
Integrity Index distribution and correlation with exam scores. Note: Figures include 44,186 schools for which the 2015 integrity index is non-missing. Panel (a) has a bandwidth of 1. Panel (b) shows the median, the 25th and the 75th percentile, the upper and lower adjacent values and outliers.

Fig. 1 presents the distribution of the integrity index in 2015 and box plots of the 2015 exam scores of schools grouped by their integrity. Fig. 1(a) confirms that cheating was widespread. Only 24 percent of schools achieved high integrity (above 80) in 2015. Moreover, a third of the schools scored below 70, which Pusmenjar uses as a threshold for sufficient evidence for cheating. This is more than in Italy and Chicago, where a similar algorithm flagged the exams of about 5 percent of classrooms as compromised (Angrist et al., 2017, Battistin et al., 2017, Jacob and Levitt, 2003), but less than in Andhra Pradesh, India, where a similar algorithm flagged 38 to 43 percent of classrooms (Singh, 2020). The integrity index was relatively constant over time, so we interpret this as a school characteristic: schools with lower integrity indices are more likely to cheat on the exam in any year. For 74 percent of schools, the difference between the 2015 and 2016 integrity index was less than 10 points.^5^ In Table A1 we show differences between schools with an integrity index above and below 70. Low integrity schools are generally smaller schools in rural areas, but teacher qualifications and the share public schools are similar between the groups.

The box plots of the exam scores in Fig. 1(b) show that the lower the integrity index, the higher the paper-based exam scores.^6^ In addition, it shows that a high school average exam score does not automatically translate into a high integrity index, meaning that the integrity index can distinguish between high scoring schools that do and do not cheat.

**Fig. 2:**
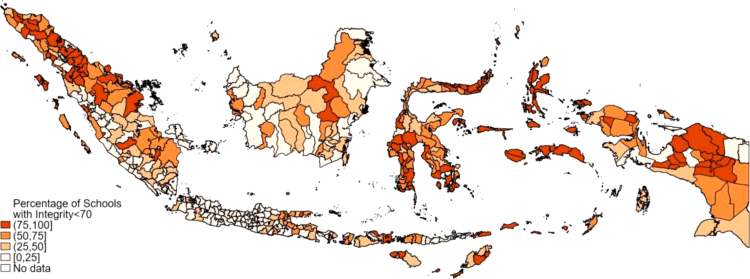
Regional variation in integrity in 2015. Note: Data shown at the district level.

There is a strong regional dimension to cheating in Indonesia. Fig. 2 shows the percentage of schools that had an integrity index below 70 in 2015 by district. Districts with many low integrity schools were often located next to each other. The regional concentration of cheating was also apparent in Italy, where most cheating took place in the southern provinces (Angrist et al., 2017).

The MoE shares the results of the integrity index with district governments to signal that they care not only about high grades on the national exam, but also about how the exam scores are achieved. However, the MoE does not implement sanctions based on the integrity index. As we discussed above, the integrity index showed little variation between 2015 and 2016. The pattern indicates that neither district governments nor schools attempted to reduce cheating in national examinations after the introduction of the integrity index.

## 3 Computer-based testing in Indonesian national examination

Prior to 2015, the Government of Indonesia had tried different policies to prevent cheating in the national exam. In 2011, the number of unique exam booklets in a room was increased from two to five. In 2013, the number was further increased to 20. In 2015, CBT was introduced with the sole aim to eradicate cheating. Students receive the exam items directly from a server with an item bank containing 30,000 items per subject. The system draws items from this bank, then gives them to the students in random order. Randomization happens both horizontally (i.e. different items for a particular competency tested) and vertically (i.e. different order of items), such that each student in the exam room has a unique test version.

The CBT prevents cheating in a number of ways. The test versions vary across students, classrooms and schools. This makes copying answers ineffective for students. In addition, neither teachers, school principals nor students have access to the test beforehand and answer sheets of the paper-based exams are useless. Finally, grading is done automatically as soon as a student completes an exam and encrypted student responses are sent directly to the central server of the MoE, so modification of the student responses by other parties is impossible.

Other aspects of CBT do not differ from paper-based testing (PBT). The paper-based and computer-based exams test the same competencies and are the same across Indonesia. The items for each of the 20 paper-based test booklets are taken from the same item bank as the computer-based test versions.^7^ In addition, both paper-based and computer-based exams are monitored by teachers from other schools in the district, who are randomly assigned by the district government. The teacher is not allowed to be in the classroom with his or her own students during the exam.

The CBT was rolled out in phases, starting from 2015 with 40 junior secondary schools. From 2016 onwards, schools could apply to participate in CBT through their district offices. Only in 30 out of 514 districts did all public schools switch to CBT in the same year, indicating that the large majority of districts adopted a gradual phase in. Schools could apply if they had access to computer facilities, internet and a stable electricity supply. Schools without the latter could apply to conduct the exam offline. Schools could also use computer facilities of a nearby school. The MoE did not provide any specific incentive or complementary inputs to schools that applied to switch to CBT. By 2019, 43,841 junior secondary schools (78%) had switched to CBT (see Table 1). Once a school switches to CBT, its integrity index can no longer be calculated, so the integrity index is set to 100.^8^

Table 1 shows the average exam score, integrity index and access to electricity, internet and computers in 2015 for all junior secondary schools, grouped by the year in which the schools switched to CBT. In the first two years, only a small percentage of schools took the CBT. The adoption of CBT began ramping up in 2017. The table confirms that adopters in the first two years are significantly different from schools that adopted CBT later or those did not adopt CBT until the end of our study period. The late CBT adopters had fewer computers, lacking electricity, and low access to internet in 2015. Schools that switched later also had lower average exam scores and integrity in 2015. In 2019, only less than a quarter of junior secondary schools (10,705 schools) still implemented paper-based exam.

**Table 1:** Staggered adoption of CBT.

	(1)	(2)	(3)	(4)	(5)	(6)
	No CBT	2015	2016	2017	2018	2019
	Mean	Difference between cohort [...] and no CBT group				
Exam score	58.68	19.71***	10.97***	3.38***	3.62***	0.87
	[12.35]	(1.45)	(1.75)	(0.97)	(0.96)	(0.80)
Within-school exam score	5.44	2.87***	3.69***	2.61***	1.14***	0.67***
Standard Deviation	[2.25]	(0.42)	(0.49)	(0.21)	(0.17)	(0.14)
Integrity index	65.62	34.38***	11.87***	9.57***	1.75	2.61**
	[17.91]	(1.04)	(1.76)	(1.24)	(1.32)	(1.14)
Exam participants	57.44	172.16***	101.91***	66.52***	22.31***	6.60**
	[67.54]	(14.32)	(15.29)	(4.52)	(4.24)	(3.11)
Student–teacher ratio^a^	13.20	3.61***	3.55***	2.73***	0.87*	−0.16
	[8.29]	(0.68)	(0.43)	(0.40)	(0.45)	(0.39)
Share teachers with 4-year degree^a^	0.82	0.09***	0.05***	0.06***	0.05***	0.04***
	[0.22]	(0.02)	(0.01)	(0.01)	(0.01)	(0.01)
Public school	0.71	0.14**	−0.31***	−0.32***	−0.42***	−0.35***
	[0.46]	(0.06)	(0.10)	(0.02)	(0.02)	(0.02)
Rural^a^	0.89	−0.69***	−0.71***	−0.41***	−0.14***	−0.09***
	[0.31]	(0.08)	(0.08)	(0.05)	(0.02)	(0.03)
Electricity^a^	0.86	0.14***	0.14***	0.14***	0.12***	0.10***
	[0.34]	(0.01)	(0.01)	(0.01)	(0.01)	(0.01)
Internet^a^	0.65	0.25***	0.32***	0.28***	0.26***	0.21***
	[0.48]	(0.05)	(0.02)	(0.02)	(0.02)	(0.01)
Computers^a^	0.19	0.74***	0.64***	0.50***	0.29***	0.17***
	[0.39]	(0.06)	(0.02)	(0.02)	(0.02)	(0.02)

Observations	10,705	40	856	9377	16,183	12,963
Cumulative (%)	21.4	0.1	1.8	20.5	52.8	78.6

Note: The table includes 50,124 panel schools for which data is available in each year between 2015 and 2019. Standard deviations are provided between brackets and standard errors between parentheses.

a We only have this information for schools that fall under the Ministry of Education. These are 33,331 schools in total. For these variables, the sample size from columns 2–6 are 39; 766; 7335; 8004; 7296, and; 9891 schools. * p < 0.10 ** p < 0.05 *** p < 0.01.

## 4 Data and descriptive statistics

We use publicly available administrative data from Pusmenjar. The data source is called Pamer (*Pengoperasian Aplikasi Laporan Pemanfaatan Hasil Ujian Nasional*) and it reports the national examination results. The dataset contains exam score means in mathematics, Indonesian, English and science, the number of students taking the exam, the standard deviations and the integrity index at the school level. We have access to mean exam scores from 2010 to 2019, standard deviations from 2010 to 2018, and the integrity index from 2015 to 2018.^9^ The exam scores are between zero and 100, and the final exam score is the average score in mathematics, Indonesian, English and science. In addition, we know which schools switched to CBT between 2015 and 2019. We complement this data with information on school resources in 2015 from MoE’s administrative data called *Dapodik* and *Sekolah Kita*. We also use the 2015 and 2018 National Socioeconomic Survey (*Susenas*), which is an annual national repeated cross-sectional household survey implemented by Indonesia’s Statistics Agency that is representative at the district level.

The exam information is available for all 56,500 junior secondary schools in Indonesia, both public and private. Private school students also take the national exam because exam scores are used in senior secondary school admission process. The data on school resources are only available for 34,412 junior secondary schools that fall under MoE. We do not have school resource information for religion-based schools under the Ministry of Religious Affairs.

**Fig. 3:**
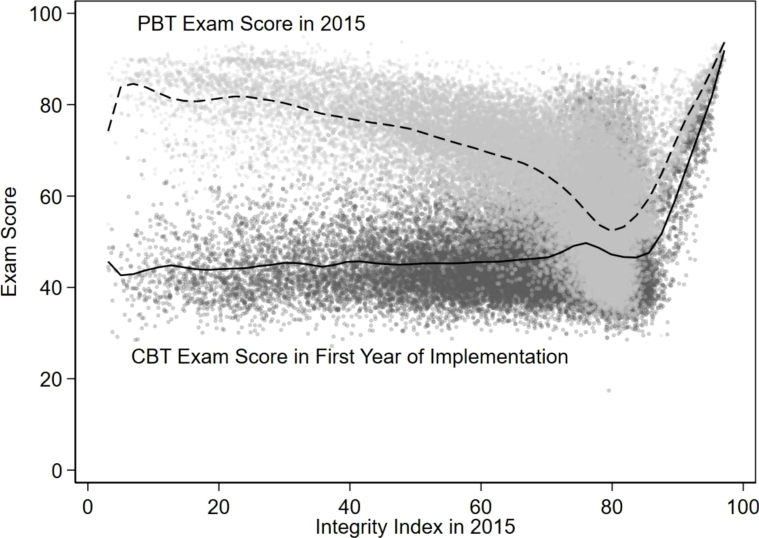
Difference between 2015 paper-based and first computer-based exam scores of treatment schools by integrity. Note: The lines represent smoothed results of a local polynomial regression. The figure includes 34,783 out of 39,379 treatment schools for which the 2015 integrity index is non-missing. The CBT score polynomial regression result combines the exam scores of all treatment schools in the first year of CBT implementation, which is between 2016 and 2019.

To get an idea of how CBT affected the exam scores, we plot the 2015 exam score and the exam score in the first year of CBT implementation as a function of the integrity score in 2015 (see Fig. 3). The dashed line indicates that in 2015, high exam scores could be obtained either through cheating or in an honest way. After switching to CBT, however, the schools that did so by cheating saw their exam score drop substantially. For schools with an integrity score below 70, the exam score dropped by 27 points on average. For honest schools we observe a much more modest drop. Note that these differences cannot be interpreted causally, because it does not correct for the general trend in exam scores over time. For instance, part of the decline in scores could be driven by changes in the difficulty of the exam. We correct for the exam score trend in the impact analysis in Section 6.

**Fig. 4:**
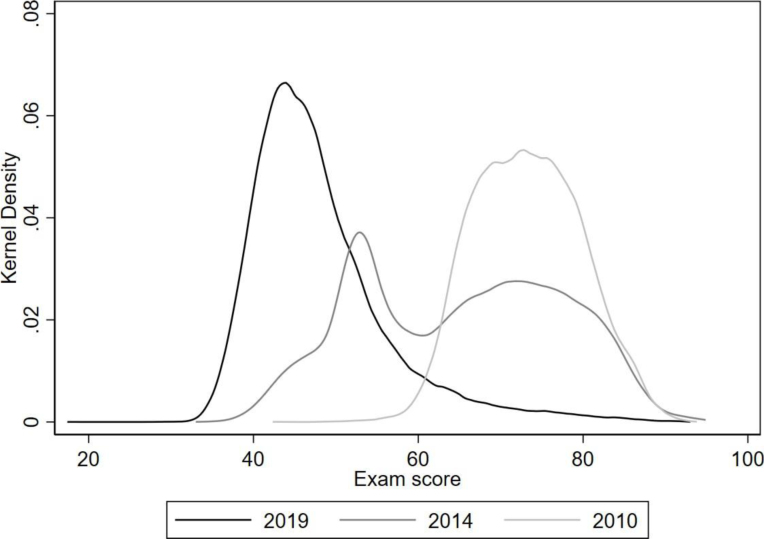
Change in exam score distribution between 2010 and 2019. Note: Kernel Distribution of exam scores by year. The figure only includes schools that had switched to CBT by 2019.

To illustrate how much cheating altered the exam score distribution, we present the exam score distribution over time in Fig. 4 for schools that had switched to CBT by 2019. As described in Section 3, in 2010, there had not been any policies against cheating yet. By 2014, the number of unique exam booklets in a classroom had increased from 2 to 20, but there was no integrity index or CBT yet. By 2019, the schools included in the figure had all switched to CBT. There is a massive shift in the distribution from a mean of over 70 points in 2010 to a mean of less than 50 points in 2019, with little overlap between the distributions. The 2014 distribution is in the middle, where we see a bump around a score of 55, the score that used to be necessary to pass the exam, and a bump above 70. The figure illustrates the extent of information distortion caused by cheating.

To assess whether the CBT exam scores capture true achievement better than PBT exam scores, we correlate CBT and PBT scores with district and school indicators for which we have a strong prior on how they are related to learning outcomes, based on the literature. For eight out of ten indicators reported in Table 2, the correlations for the CBT exam scores are more closely aligned with expectations than for the PBT score. For example, a 10 percentage point higher share of the population that is literate is associated with 1.603 points higher average exam score when done using CBT but associated with 1.209 points lower average exam score when done using PBT. Only for average district years of schooling, and the indicator for schools in rural areas, the correlation with CBT scores is not more aligned with expectations than the correlation with PBT scores. However, in both cases, the difference in correlation is statistically insignificant.

While there seemed to be no socioeconomic gap in exam scores under the PBT exam, CBT revealed that there was a gap. Between 2015 (PBT) and the first year of CBT implementation, the gap in average exam scores between schools in the 20 percent poorest and 20 percent wealthiest districts increased from 0.5 points to 6.2 points when based on expenditure per capita, and from −1.9 (the poorest districts scored higher) to 4.6 points when based on the poverty rate (numbers not shown in the table). This is important information for the government when deciding where to allocate resources.

The implementation of CBT was accompanied by a stark reversal of the rankings across schools and districts. Table 3 presents the rank correlations of the average exam score between 2015 and other years (4 years before and 4 years after 2015) at the school and district level. The table includes 226 out of 514 districts in which all schools implemented CBT by 2019, so the rank correlation between 2015 and 2019 provides an indication of how different school and district ranks were with and without cheating.^10^ The rank correlations between 2015 and earlier years show whether these ranks also differed across years when cheating was still possible, and the rank correlations between 2015 and 2018 show the gradual change in ranks as more schools in the districts switched to CBT.

**Table 2:** Correlations between exam scores and district and school-level indicators by test taking method.

	PBT	CBT	Difference
	2015	in 1st year of implementation	(*p-value*)
*District indicators*			
Average years of schooling	1.17	0.85	0.61
	(0.05)***	(0.03)***	
Share of population that went to Preschool	5.54	7.69	0.68
	(0.49)***	(0.30)***	
Share of population that is literate	−12.09	16.03	0.07
	(1.33)***	(0.67)***	
Net enrollment in junior secondary school	11.08	14.22	0.76
	(1.18)***	(0.69)***	
Log expenditure per capita	2.18	5.29	0.20
	(0.25)***	(0.19)***	
Share of population that is poor	4.39	−23.59	0.04
	(1.37)***	(0.83)***	
Share of population with internet access	4.91	13.17	0.07
	(0.48)***	(0.34)***	
*School indicators*			
Share teachers with 4-year degree	1.83	4.10	0.05
	(0.51)***	(0.37)***	
Rural school	−4.58	−3.08	0.20
	(0.18)***	(0.14)***	
Internet access	0.44	1.35	0.11
	(0.22)**	(0.13)***	
Year fixed effects	Yes	Yes	

Observations	39,379	39,379	

Note: District indicators come from Susenas 2018 and school indicators from Dapodik 2015 and 2018 (except for the number of exam participants which we have for each year). Table includes PBT scores in 2015 and the CBT scores in the first year of implementation for the 2017, 2018 and 2019 CBT cohorts, as in Fig. 3. Each correlation is estimated separately because of strong correlations between indicators, and is corrected for year fixed effects in exam scores. Standard errors are corrected for clustering at the district level. * p < 0.10 ** p < 0.05 *** p < 0.01

The first column looks at the school percentile correlation across all 226 districts. In years before the start of the CBT program in 2015, the rank correlation varied between 0.45 and 0.61 with higher rank correlations closer to the base year. In the years after the start of the CBT program, the same pattern is observed but the rank correlation dropped to 0.18 in 4 years. Column 2 presents the average decile rank correlations of schools within districts. We take decile ranks because of the large variation in the number of schools within districts (minimum 11, maximum 966). Interestingly, the opposite pattern arises. Average rank correlations after 2015 are somewhat higher than they were before CBT started indicating that school quality is the main determinant of school ranks within districts. On the other hand, the rank correlations across districts dropped sharply after implementation of CBT. While the rank correlation was in the range of 0.53 to 0.63 before the start of CBT, it turned even negative in years thereafter. The evidence shows that the loss in rank correlation is mostly resulting from rank reversals across districts, and less so from rank reversal of schools within districts. This is in accordance with the findings in Fig. 2, that shows that cheating in concentrated at the regional level.

**Table 3:** Rank correlation over time for districts with full CBT implementation by 2019.

	(1)		(2)		(3)
	School percentile		School decile within district		District rank
2011	0.45		0.63		0.53
2012	0.50		0.66		0.59
2013	0.59		0.74		0.58
2014	0.61		0.76		0.65
2015	1		1		1
2016	0.67		0.82		0.67
2017	0.48		0.75		0.51
2018	0.23		0.65		0.06
2019	0.18		0.68		−0.1

Observations	24,028		24,028		226

Note: Table shows the Pearson pairwise correlation coefficient of the (percentile) rank in each year with the (percentile) rank in 2015. It includes 24,028 panel schools from 226 (out of 514) districts in which all schools implemented CBT by 2019. None of these schools implemented CBT in 2015, 3 percent in 2016, 35 percent in 2017 and 80 percent in 2018. The median percentage of schools in the district that implemented CBT gradually increased from 5 percent in the first year, to 33 percent in the second year, 76 in the third year and 100 percent in the fourth year. There are between 11 and 966 schools in a district (109 on average).

## 5 Empirical strategy

To measure the impact of CBT on test scores, we conduct a series of difference-in-difference (DiD) estimations. We estimate the impact separately for each group of schools that switched to CBT in a different year, which we call treatment cohorts. We perform a separate DiD estimation for each CBT cohort because recent studies find that treatment effect estimators from two-way fixed effects models (period and group fixed effects) are biased when there are heterogeneous and dynamic treatment effects. These studies show that the coefficients are weighted sums of the average treatment effects across cohorts and the weights of some cohorts could be negative (Goodman-Bacon, 2021, Callaway and Sant’Anna, 2021, De Chaisemartin and D’Haultfoeuille, 2020, Sun and Abraham, 2021). Because of the integrity differences between the CBT cohorts, we expect heterogeneous treatment effects. Following the recent literature, we estimate the average treatment effect for each cohort separately.

To estimate a combined effect across cohorts, we use the identification strategy of Callaway and Sant’Anna (2021) for staggered DiD models with variation in treatment timing. From the growing literature on DiD models with two-way fixed effects, the Callaway and Sant’Anna estimators suit our study best. Their method allows for dynamic effects when the treatment is binary and the design is staggered. We expect dynamic effects over time as schools might try to improve their exam scores again after experiencing a drop. Like all recently suggested estimators for DiD models with two-way fixed effects, they remove the inappropriate comparison schools from the control group. A standard two-way fixed effect model includes all schools that did not switch treatment status in the comparison group, including schools that remained treated. In case of dynamic effects, this creates a bias in the estimates as the common trend assumption does not hold. We decide to use the same control group for all schools. We use the never-treated schools, which still used paper-based exams in 2019, as the comparison group for each treatment cohort.^11^ The results can be interpreted as a sample-weighted average treatment effect on the treated schools.

We allow for one period of anticipation of the treatment, meaning that we use the second-to-last year before treatment implementation as the base period to estimate the treatment effects. Anticipatory behavior could occur if treatment schools knew in advance the year they would switch to CBT. The treatment schools might want to limit the drop in exam scores once they implement CBT by already reducing cheating practices in year prior to implementation. As shown later in the results, we indeed find evidence for small anticipation effects on the last year before switching to CBT.

We are interested in the effect of CBT on school mean exam scores and the spread of the exam scores within schools.^12^ We estimate the following model for each of the treatment cohorts separately using data between 2015 and 2019,

(1) $$Y_{sdt}=\alpha_{t}+\alpha_{s}+\overset{-3}{\underset{e=-K}{\sum }}\delta_{e}\cdot D_{sdt}^{e}+\delta_{-1}\cdot D_{sdt}^{-1}+\overset{L}{\underset{e=0}{\sum }}\beta_{e}\cdot D_{sdt}^{e}+\epsilon_{sdt}$$

where Y is the average exam score or the standard deviation of student exam scores within school s, district d at time t, $\alpha_{t}$ and $\alpha_{s}$ are time and school fixed effects^13^, respectively, $D_{sdt}^{e}$ are indicators for a school s being e periods away from initial treatment at time t, and $\epsilon_{sdt}$ is the error term. K and L are the earliest and latest period in the data available for a specific cohort, which are 2010 and 2019, respectively. Since we allow for one period of anticipation, the coefficients are relative to period e=−2, or the second to last period before treatment. The parameters of interest are $\beta_{e}$, measuring the effect of participating in CBT at period e. In the years that the treatment schools implement CBT, we expect β to be negative for the school mean exam scores and positive for the within school standard deviation of the exam scores. The standard errors are computed using multiplier bootstrap (Callaway and Sant’Anna, 2021) and are corrected for clustering at the district level. We also present aggregated treatment effects, which are sample-weighted average treatment effects across cohorts in each period relative to period e=−2, for periods for which we have estimates for all cohorts.

The model is estimated on a balanced sampled of schools for which we have complete exam data for each year between 2010 and 2019.^14^ We exclude schools that switched to CBT in 2015 or 2016 from our analysis (2 percent of treated schools). These schools had significantly different characteristics from the relevant comparison group, so the common trend assumption is unlikely to hold. The 2015 cohort was a pilot cohort of only 40 schools. The 2016 cohort has a much higher average integrity score and higher average exam scores than the comparison group (about 10 points, see Table 1). Hence, it has little common support with the comparison group. In Figure A3 we show the propensity to be treated for each cohort, based on a logit model of CBT implementation on the exam scores between 2010 and 2015 and the integrity score in 2015. We find that there is substantial overlap for schools that switched to CBT in 2017 and later, and show the lack of common support for the 2016 cohort. We also remove 188 schools from our sample that switched back from CBT to PBT before 2019 as De Chaisemartin and D’Haultfoeuille (2020) point out that the weighted average of the cohort-specific treatment effects is only valid when treated groups remain treated after their first year of treatment implementation. The analysis sample contains 39,420 panel schools out of 56,242 schools that took the exam in 2019. Analyses for which we use the integrity index or school characteristics have fewer observations, because the integrity index is only available for 35,493 out of the 39,420 schools and school characteristics are available for 26,662 schools.

The causal interpretation of our results depends on two important assumptions. First, we assume that the average student ability within schools is stable over time. Each year a different group of students took the exam. We are only able to attribute a difference in exam scores over time at the school level to CBT if the underlying ability of the students remained the same. The assumption would be violated if students changed schools because of CBT. We argue that this is unlikely. Students enrolled three years before they took the exam, so they could not anticipate whether their school would opt into CBT. Also, CBT adoption increased rapidly between 2017 and 2019, making strategic decisions by parents less likely. The assumption would also be violated if weaker students were not allowed to take the exam. This is unlikely to happen because, while students must take the exam to graduate, their performance in the exam does not determine their graduation. Therefore, schools do not have any strong incentive to hold students back, especially since parents want their children to graduate on time. To assess whether the students composition changed due to CBT, we estimate the impact of CBT on the number of exam participants of each school and find no statistically significant effect (see Figure A4).^15^

The second assumption is the common trend assumption. We assume that the trend in exam scores of the treatment and comparison group would have been the same if the exams would have remained on paper. We test for differences in the pre-treatment exam score trend between the treatment and comparison group in Eq. (1) with estimates for each of the pre-treatment periods, i.e. $\delta_{e}$. We interpret similar pre-trends as supporting evidence that the common trend assumption will also hold in post-treatment periods.

We present heterogeneous treatment effects by subject, the schools’ integrity level in 2015, and by whether the school had access to computers in 2015.^16^ With this heterogeneity analysis we test whether the effects we observe from CBT are indeed resulting from a reduction in cheating, and not from other factors associated with the method of exam taking. If the effect is due to a reduction in cheating, it should be larger for schools with a low integrity score. On the other hand, if it is due to students being unfamiliar with working on computers, the effect should be smaller for schools that already had computers in 2015 and should be similar across subjects. We conduct a decomposition analysis to delve deeper into this issue.

Spillovers could arise if the roll-out of CBT in a district results in a norm change with respect to the acceptability of cheating in the national exam. For example, exam supervision is organized by district governments, which allocate teachers from different schools as proctors to supervise exams. If these proctors came from schools that switched to CBT, they may have been stricter than usual because their school had no option to cheat anymore. Allowing the other school to cheat would lead to increase the chance that their own school rank lower, hence losing prestige. In addition, the distribution of answer sheets among students and teachers may have been disrupted because the answer sheets are of no use to the ones that took the exam on computers. This way, the probability that a student that took the exam on paper acquiring an answer sheet becomes smaller as more schools switch to CBT. To investigate whether the spillover hypothesis is true, we estimate Eq. (2) using data from comparison schools only

(2) $$Y_{sdt}=\alpha_{t}+\alpha_{s}+\delta_{1}\cdot {\bar{D}}_{dt}+\epsilon_{sdt}$$

where Y is the mean exam score or the integrity score in the years 2015 to 2019 of the schools that had not yet implemented CBT by 2019. ${\bar{D}}_{dt}$ is the fraction of schools that implemented CBT in district d in year t.

After establishing the presence of spillover, We perform two corrections that adjust the main estimates for these potential spillover effects. First, we correct the comparison group trend in exam scores for the decline in cheating using the integrity index. This robustness check corrects the estimates for a change in cheating practices in comparison schools directly. However, since we do not have access to the 2019 integrity score, we can only apply this correction for the cohorts that switched to CBT in 2017 and 2018. We correct the main estimation as specified in Eq. (1) by holding the integrity index of the comparison schools constant. We estimate the following equation

(3) $$Y_{sdt}=\alpha_{t}+\alpha_{s}+\overset{-3}{\underset{e=-K}{\sum }}\delta_{e}\cdot D_{sdt}^{e}+\delta_{-1}\cdot D_{sdt}^{-1}\;+\overset{L}{\underset{e=0}{\sum }}\beta_{e}\cdot D_{sdt}^{e}+\theta_{1}\cdot \left(1-D_{sdt}^{L}\right)\times I_{sdt,t\in 2015-2018}+\epsilon_{sdt}$$

which is the same as Eq. (1), but with the addition of integrity index $I_{sdt}$ interacted with a dummy variable that indicates comparison schools which did not implement CBT in 2019 ($D_{sdt}^{L}=0$). The integrity index is only available from 2015 until 2018, as indicated by t∈2015−2018 in the subscript, so the correction term $\theta_{1}\cdot \left(1-D_{sdt}^{L}\right)\times I_{sdt,t\in 2015-2018}$ is zero in other years.

Second, we apply a similar correction but now use the share of schools in the district that switched to CBT to capture the spillover effects. We can apply this robustness check to all treatment cohorts and all years. We estimate the following model, allowing the test scores of comparison schools to vary with the share of schools that switched to CBT,

(4) $$Y_{sdt}=\alpha_{t}+\alpha_{s}+\overset{-3}{\underset{e=-K}{\sum }}\delta_{e}\cdot D_{sdt}^{e}+\delta_{-1}\cdot D_{sdt}^{-1}+\overset{L}{\underset{e=0}{\sum }}\beta_{e}\cdot D_{sdt}^{e}+\delta_{1}\cdot \left(1-D_{sdt}^{L}\right)\times {\bar{D}}_{dt}+\epsilon_{sdt}$$

This is the same equation as Eq. (3), but we replace $I_{sdt}$ with ${\bar{D}}_{dt}$. We basically combine Eq. (1) and Eq. (2) in Eq. (4) because we only allow the comparison group trend to vary with the share of CBT in the district. Conditioning on the integrity index or CBT implementation in the district, we expect a larger negative treatment effect because we hypothesize that comparison schools in districts with a higher fraction of treated schools have a more downward trend in exam scores.

One could be concerned about reverse causality between the share of schools implementing CBT in the district and cheating in PBT schools. For instance, in districts with more cheating, less schools might be inclined to participate in CBT. Our estimates correct for this type of selection using school fixed effects which also capture pre-2015 levels of cheating. As an additional robustness check, we correct the estimates for linear district trends in exam scores. These correct for potential dynamic factors which could affect both CBT adoption and cheating in PBT schools. These could occur if district governments that are motivated to restrict cheating could pressure schools to transition to CBT and reduce cheating on PBT exams at the same time. We argue that if this is the case, then the drive to reduce fraud would have already been present before CBT implementation, and this attitude would present itself in a decreasing exam score trend. We add linear district trends to our main model to allow for this possibility, where $\gamma_{d}$ are district indicators and t is the year.

(5) $$Y_{sdt}=\alpha_{t}+\alpha_{s}+\overset{-3}{\underset{e=-K}{\sum }}\delta_{e}\cdot D_{sdt}^{e}+\delta_{-1}\cdot D_{sdt}^{-1}+\overset{L}{\underset{e=0}{\sum }}\beta_{e}\cdot D_{sdt}^{e}+\gamma_{d}+\gamma_{d}\cdot t+\epsilon_{sdt}$$

## 6 Results

### 6.1 School average exam scores

**Fig. 5:**
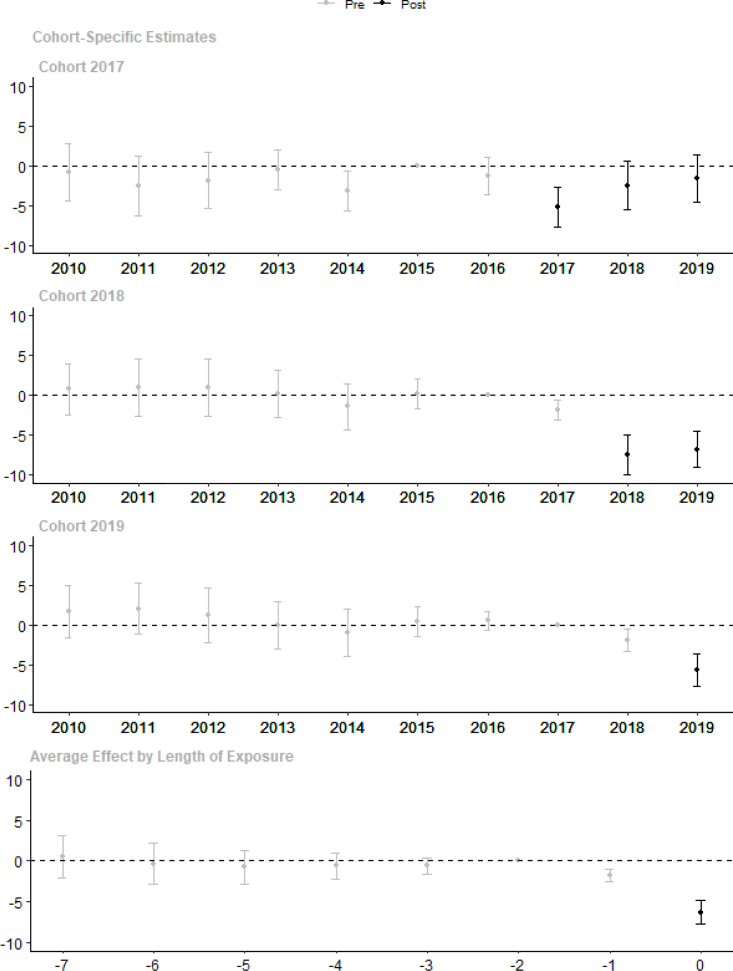
Impact Estimation Result on School Exam Scores. Note: The 2017 cohort includes 15,468 panel schools, the 2018 cohort 20,151 schools and the 2019 cohort 17,102 schools. Plot of post-CBT point estimates of $\beta_{e}$ and pre-CBT point estimates of $\delta_{e}$ in Eq. (1) with 95% confidence interval, estimated separately for each cohort. Standard errors are corrected for clustering at the district level. The ’average effect by length of exposure’ figure shows the sample-weighted average effect across cohorts. Detailed results are available in Table A3.

Fig. 5 plots the estimated treatment effect in each year for each cohort (the detailed regression results can be found in Table A3). The pre-intervention trend estimates confirm that the effect arises in the year of opting in. We only find one coefficient that is significantly different from zero between the comparison group and the 2017 cohort in the pre-trend. In Section 6.4, we show that our results are robust against conditioning on school characteristics and on pre-trend scores, confirming that this minor pre-trend difference is not a concern for our analysis.

The combined estimates also show a small anticipation effect of 1.7 points in the year prior to CBT implementation. In Figure A5 we show that the pretrends in terms of the integrity index, however, do not differ significantly between the treatment and comparison group. This suggests that anticipation of CBT implementation by changing cheating practices is a minor concern.^17^

CBT resulted in a drop of 6.3 points in school average exam scores in the first year of implementation. The effect is larger for the 2018 cohort (7.5 points) than for the 2017 and 2019 cohort (5.2 and 5.7 points respectively). This makes sense because the 2018 cohort’s integrity index is lower than that of the other cohorts (see Table 1). The combined effects, presented in the last row of Fig. 5, are the sample weighted averages of the cohort effects. We only present results for the first year of implementation because that is the only year in which all cohorts feed into the estimates. Estimates in later implementation years that include less than three cohorts would be subject to selection bias.

Fig. 5 also shows the effects in later implementation years for the 2017 and 2018 cohorts. Although the results for the 2017 cohort suggests that the impacts declined over time, they are not robust to the spillover correction, as shown later in Fig. 9.

Table A4 presents the impact estimation results on exam scores in terms of standard deviations. We used the within-school standard deviation of the test scores, the school level mean exam scores and the number of students that took the exam to calculate the student level mean and standard deviation of the comparison group exam scores in each year and used these to standardize the exam scores.^18^ Average school level exam scores drop with 0.5 standard deviation in the first year of CBT implementation.

**Fig. 6:**
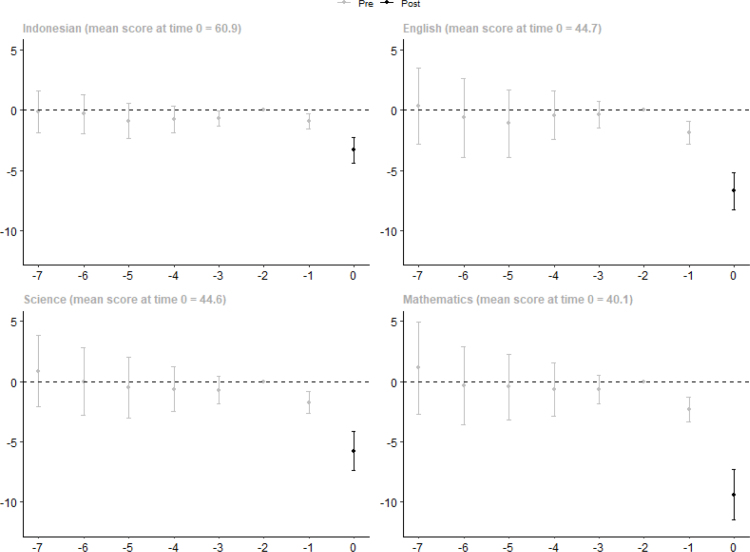
Impact estimation result on school exam scores by subject. Note: Plot of post-CBT point estimates of $\beta_{e}$ and pre-CBT point estimates of $\delta_{e}$ in Eq. (1) with 95% confidence interval. Figure shows the average effect by length of exposure, which is the sample-weighted average effect across cohorts. Standard errors are corrected for clustering at the district level. Detailed results are available in Table A5.

We next present average heterogeneous effects by subject, integrity score and availability of a computer lab. We show first year average effects by subject in Fig. 6.^19^ Effects are larger in subjects in which the average exam score in the first year after switching to CBT were lower. This suggests that there was more cheating on subjects that students found more difficult.^20^

**Fig. 7:**
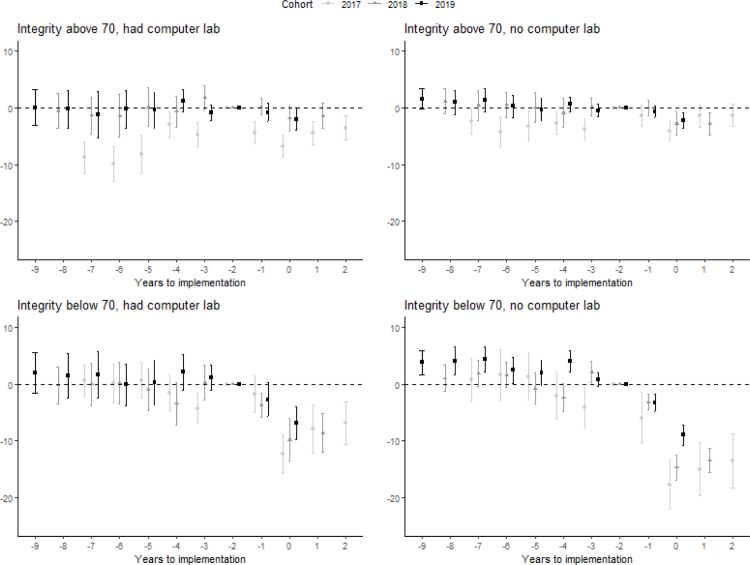
Impact estimation result on school exam scores by baseline integrity and computer ownership. Note: Plot of post-CBT point estimates of $\beta_{e}$ and pre-CBT point estimates of $\delta_{e}$ in Eq. (1) with 95% confidence interval, estimated separately for each cohort and integrity and computer ownership category. The figure includes 30,198 schools for which the integrity index and computer information is available in 2015. The integrity categories are based on the integrity index in 2015. Standard errors are corrected for clustering at the district level. Detailed results are available in Table A6.

Fig. 7 plots the estimates separately for schools with an integrity index below 70 and above 70, and with and without computers in 2015. The latter is to test whether the effect is likelier due to a decline in cheating rather than a lack of computer skills. We show cohort-specific estimates because we find diverging pre-trends for schools in the 2017 cohort with an integrity index above 70, and for schools in the 2019 cohort with an integrity index below 70 and without a computer lab. Although we are careful with interpreting these results, the similarity across cohorts in the effects in each group provides compelling evidence that the effect is driven by a reduction in cheating rather than a lack of computer skills.^21^ We show heterogeneity results only by the integrity index in Figure A6.

Schools with low integrity and those without computers in 2015 were more affected by the switch to CBT. The effect of integrity is much larger than the effect of having computers, indicating that the effect of the CBT mainly operated through a reduction in cheating rather than through the change in test taking mode (from paper to computers).^22^ For low integrity schools with a computer lab in 2015, CBT resulted in a drop in exam scores between 6.8 points (2019 cohort) and 12.3 points (2017 cohort) while for high integrity schools the drop was only 2.1 and 6.8 points for the corresponding cohorts, respectively. Not having computers resulted in a 5-point larger drop for low integrity schools in the 2017 and 2018 cohorts and a 2-point larger drop for the 2019 cohort, but a maximum effect of 2 points for high integrity schools, indicating that for the latter group, familiarity with computers did not drive the small decrease in exam scores.^23^

To assess the relative contribution of access to computers and cheating to the drop in test scores as a result of CBT, we predict the drop in test scores in the first year in which CBT was implemented using the estimates shown in Fig. 7 (Table A6). Averaging over the cohorts, we predict a drop in 6.6 points (not reported in the table). To assess the role of computers, we apply the parameters of the schools with computer labs for the schools that had none, separately for low and high integrity schools. This yields a drop of 5.8 points. In a similar fashion, to assess the role of cheating we use the parameters of the schools with high integrity scores for the schools with low integrity scores. This yields a predicted drop of 3.4. The results of the exercise indicates that while both access to computers and cheating contributed to the drop in test scores resulting from CBT, the drop due to cheating is about four times larger.^24^

### 6.2 Spread of the exam scores within schools

**Fig. 8:**
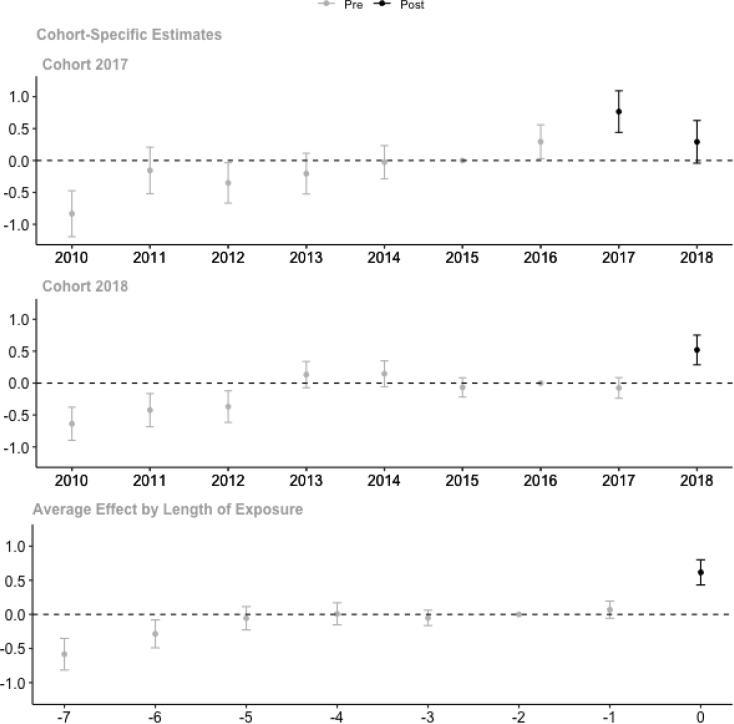
Impact estimation result on standard deviation of exam scores within schools. Note: Plot of post-CBT point estimates of $\beta_{e}$ and pre-CBT point estimates of $\delta_{e}$ in Eq. (1) with 95% confidence interval, estimated separately for each cohort. Standard errors are corrected for clustering at the district level. Figure shows the average effect by length of exposure, which is the sample-weighted average effect across cohorts. The year 2019 is not included in the figure because the within-school standard deviation of the exam scores is not available in that year. Detailed results are available in Table A7.

If cheating is eliminated, the exam results can more accurately distinguish students in terms of their abilities. We expect the exam results distribution in a school to be larger after the switch to CBT. As expected, the standard deviation of (raw) exam scores within schools increased with 0.8 and 0.5 for the 2017 and 2018 cohorts, respectively, when these schools switched to CBT (Fig. 8). The detailed regression results can be found in Table A7 in Appendix. As described before, it is likely that lower-performing students benefited more from cheating before CBT. Figure A7 shows this pattern descriptively, with a larger increase in the within-school standard deviation of exam scores for schools with lower integrity scores in 2015.

The disappearance of the treatment effect on the standard deviation in the second year of CBT implementation for the 2017 cohort suggests that lower-performing students improved their test scores more than higher-performing students. However, these results should be interpreted carefully because the trends of the treatment and comparison groups are only parallel from 2013 onwards.

### 6.3 Local spillovers of CBT

To investigate the spillovers, we first look at the correlation between a change in the fraction of schools in the district that implemented CBT and a change in exam scores and the integrity index of the comparison schools, for which we estimate Eq. (2). The results are shown in Table 4, Table 5. We look at the district level, because education policy is determined at that level and proctors are assigned to schools within the district. Recall that it was not whole districts that switched, but schools within districts opted in. The average district has 107 junior secondary schools.

The more schools in a district switched to CBT, the lower the exam scores of the comparison schools (Table 4) and the higher their integrity (Table 5). Only the exam scores of schools with integrity below 70 significantly decreased as more schools in the district switched to CBT, suggesting that the exam score difference was due to a reduction in cheating practices.

Consistent with the hypothesis that cheating reduced among comparison schools, we find that the integrity index of comparison schools was larger as CBT implementation around them increased in Table 5. We expect a larger scope for spillover effects in districts with many cheating schools. Moreover, if the spillover effects represent a decrease in acceptability of cheating when the extent of cheating in the district declined, we would only expect to find spillover effects from initially cheating schools that switched to CBT. These hypotheses are confirmed by the results in the table.

**Table 4:** Correlation between a change in the fraction CBT in district and a change in the exam scores of comparison schools.

Dependent variable:	(1)	(2)	(3)
Exam score	All	Integrity < 70	Integrity >= 70
Fraction CBT in the district	−1.65	−9.78	0.83
(excluding the observed school)	(1.77)	(2.77)**	(1.51)
School fixed effects	Yes	Yes	Yes
Year fixed effects	Yes	Yes	Yes

Observations	53,525	21,360	25,655
Number of schools	10,705	4272	5131

Note: Model specified in equation 2, estimated on the comparison schools. Standard errors between parentheses and corrected for clustering at the district level. Each regression includes year and school fixed effects. Column 2 and 3 have fewer observations than column 1 due to missing values of the integrity index. * p < 0.10 ** p < 0.05 *** p < 0.01.

Anecdotal evidence suggests that various mechanisms are at play. The change could arise from the fact that exams are proctored by teachers from other schools in the same district. Teachers from schools that switched to CBT may have become stricter when proctoring schools that conduct paper-based exams to ensure fair competition. Another reason could be that honest students and teachers in paper-based exam schools were under less pressure to cheat as scores in nearby CBT schools declined. Finally, with more schools participating in CBT, exam answer keys are harder to acquire. Data availability limits our scope to test the importance of these which of these potential mechanisms.

**Table 5:** Correlation between a change in the fraction CBT in district and a change in the integrity index of comparison schools.

Dependent variable:	(1)	(2)	(3)	(4)
Integrity index	All	Share cheating in	Share cheating in	
		district < 0.5	district >=0.5	
Fraction CBT in the district	10.34	2.40	21.44	
(excl observed school)	(2.87)***	(1.69)	(5.72)***	
Fraction CBT among cheaters				17.98
				(8.23)**
Fraction CBT among non-cheaters				2.51
				(6.90)
School fixed effects	Yes	Yes	Yes	Yes
Year fixed effects	Yes	Yes	Yes	Yes

Observations	40,352	22,069	18,277	18,188
Number of schools	10,671	5824	4844	4844

Note: Model specified in equation 2, estimated on the comparison schools. The integrity index is not available in 2019. Standard errors between parentheses and corrected for clustering at the district level. Each regression includes year and school fixed effects. * p < 0.10 ** p < 0.05 *** p < 0.01.

### 6.4 Robustness checks

**Fig. 9:**
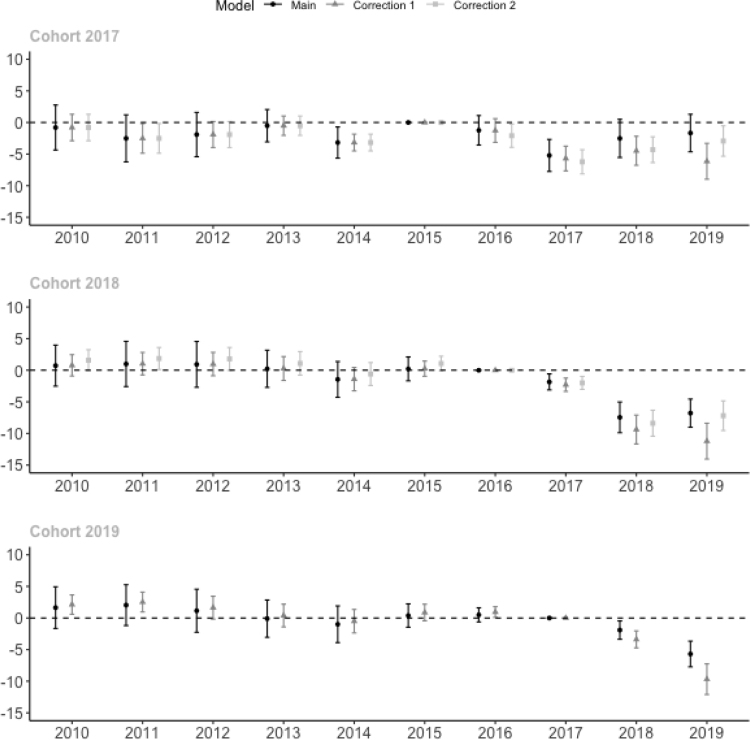
Impact estimates on exam scores corrected for spillover effects on comparison schools. Note: Correction 1 corrects comparison school scores for the change in the share of CBT in the district. Correction 2 corrects comparison school scores for the change in their integrity index. Model specified in Eqs. (3) and (4). We do not implement Correction 2 for the 2019 cohort because it cannot correct the impact estimate in 2019 for the change in integrity among comparison schools between 2018 and 2019. Main results are from Fig. 5. Table includes panel schools that participated in the exam each year between 2010 and 2019. Standard errors between parentheses and corrected for clustering at the district level. Each regression includes year and school fixed effects. The integrity index is unavailable in 2019.

The spillover effects reported in the previous section affect the interpretation of the results reported in Section 6.1. In Fig. 9, we present the results for the impact of CBT on exam score (previously reported in Fig. 5 and Table A3) with the correction for spillover effects as discussed in Eqs. (3) and (4). The correction barely affects the pre-trend coefficients as presented in Fig. 5 because there was only little CBT implementation in 2015 and 2016 and we do not have integrity scores before 2015.

As expected, the impact estimates increase in size when we correct for spillover effects. Correcting for the decline in cheating amongst comparison schools using CBT implementation makes the biggest difference to our estimates. Because a larger faction of schools switched to CBT as time passes, the later years are affected more. As a result, the declining trend in impact of CBT which was observed for the 2017 cohort is no longer visible with the correction for spillovers.

Overall, the robustness checks make little difference because even though we find evidence for spillover effects, the correlation between the fraction of schools in the district that implement CBT and the average PBT exam score is small and insignificant when estimated on the full sample of schools (column 1 of Table 4). In addition, the coefficients shown in Table 4 should be interpreted as the difference in the average exam score or integrity index when CBT implementation among other schools in the district increases from 0 percent of schools to 100 percent. The yearly increase in CBT implementation is smaller than that. On average, 1.4 percent of schools in each district implemented CBT in 2016, 19 percent in 2017, 46 percent in 2018 and 70 percent in 2019. This change is too small to generate spillover effects that substantially affect our estimates. Hence, our impact estimates are robust against controlling for the spillover effects.

**Fig. 10:**
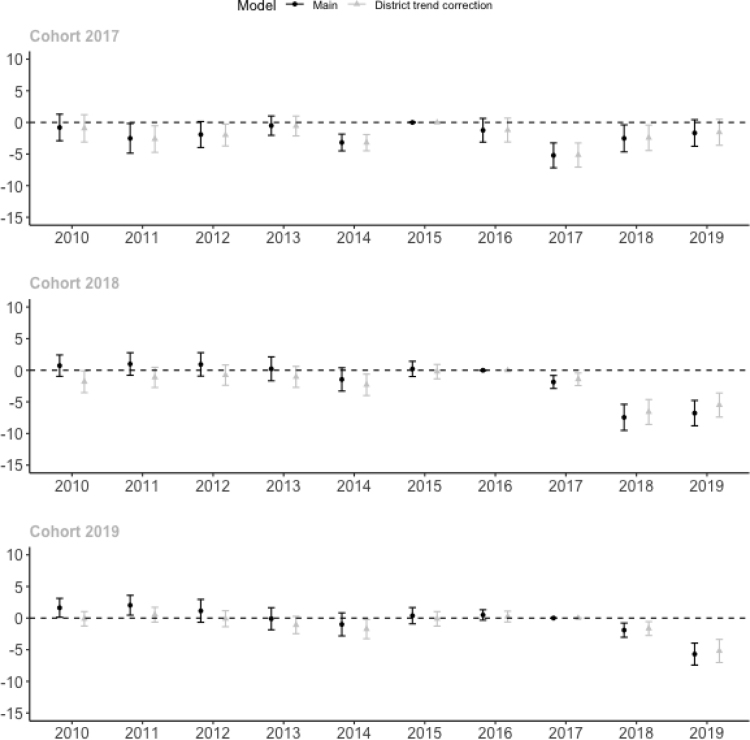
Impact estimates on exam scores corrected for district trends. Note: Correction adds linear district time trends to the main model, see Eq. (5). Main results are from Fig. 5. Table includes panel schools that participated in the exam each year between 2010 and 2019. Standard errors between parentheses and corrected for clustering at the district level. Each regression includes year and school fixed effects.

To see whether district attitudes towards cheating may explain the correlation between the share of CBT in the district and the decline in PBT exam scores, we correct the estimates for linear district trends in exam scores as specified in Eq. (5). District governments that are motivated to restrict cheating could pressure schools to transition to CBT and reduce cheating on PBT exams at the same time. However, we find that our results are also robust against including district linear trends in exam scores in our model (see Fig. 10).

Finally, as mentioned above, to provide additional evidence for the validity of the common trend assumption, we perform two additional robustness checks. First, in Figure A8, we estimate the results conditional on school characteristics in 2015, specifically, exam score, integrity index, number of students, student-teacher ratio, public school, share of teachers with 4-year degree, rural, electricity access, internet access, computer availability and island group. Second, in Figure A9, we estimate the results conditional on the pre-trend between 2010 and 2015. The common trend assumption is less strict for these estimates, as we only assume that schools with the same characteristics would follow the same exam score trend in the absence of CBT. We use the doubly robust estimation procedure as proposed by Callaway and Sant’Anna (2021). The impact estimates are similar to our main results, showing that the minor pre-trend differences found in Fig. 5 are not a concern for the interpretation of our results.

## 7 Conclusion

Cheating on examinations is costly to exam takers and society because it distorts the signaling value of the exam results. Student performance on exams is used by higher education institutions and employers to assess the quality of their applicants, and by policymakers to evaluate school quality. In case of widespread cheating, it becomes difficult for high-ability students to signal their skills. Education institutions and employers might therefore use noisy signals of applicant ability, such as the reputation of the school from which the applicant graduated. This likely hinders education opportunities for the poor, who often lack access to the schools with the best reputation. Moreover, with widespread cheating, policymakers do not know where school quality is lacking. To illustrate, we find no socioeconomic gap in PBT exam results, while the CBT exam results reveal a gap of about 6 points between the bottom and top 20 percent wealthiest districts. Policymakers might not allocate resources to the places that need them most in that case, likely hurting poorer students as well.

In Indonesia, anecdotal evidence about widespread cheating in Indonesia’s national examinations had existed for decades. Early efforts to reduce cheating, such as increasing the number of test booklets, did not reduce the problem most likely because, in the presence of high rates of cheating, both educators and students had an incentive to continue to participate in this practice. To measure the magnitude of the problem, the MoE developed an integrity index to detect cheating. In 2015, 33 percent of the junior secondary schools had an integrity index below 70, a threshold that indicates strong evidence of cheating.

To prevent cheating, the MoE decided to use CBT for the national exam starting in 2015. The implementation was gradually phased in, allowing us to estimate the impacts of switching to CBT using DiD. We present effects for the 2017, 2018 and 2019 cohorts, which together represent 77 percent of all junior secondary schools.

We find that CBT caused a substantial decline in scores. Exam scores decreased by 6.3 points on a 1–100 scale, equivalent to 0.5 standard deviations, and the within-school spread of exam scores increased by 0.6 standard deviations. Among schools for which the integrity index indicated a high likelihood of cheating, the drop in exam scores was in the 6.8–12.3-point range. By comparing the treatment effects on high and low integrity schools and schools with and without computers in 2015, we confirm that the decline in exam scores was mainly driven by a reduction in cheating. If it had been the test taking technology that caused the drop in scores, we would expect to see smaller effects in schools which had access to computers. We observe no such effect. Further, we observe stronger impacts for more difficult subjects for which the payoff of cheating was higher.

We also find that the phase in of CBT at the district level reduced cheating in schools still conducting the paper based exam. While we cannot test for the mechanisms behind this finding, we believe it is indicative of a change in norms. When the schools that adopt CBT have to play by the rules, they may assert pressure on other schools in the same district to do so as well. It may also reflect a change in the logistics of cheating. With more schools switching to CBT, the demand and supply of answer keys are lower. However, the spillover effects are small. Correcting for them does not substantially alter the conclusions with respect to the immediate effect of CBT on test scores.

We find that the impacts are persistent over time. Because the MoE canceled the national exams since 2020, we could only analyze the impacts for a maximum of three years. There was no clear upward or downward trend in the point estimates, and they are usually statically indistinguishable. For most groups, except of the schools with high integrity and access to computers, the effects remain significantly different from zero. On the one hand, this is positive news in the sense that it indicates that stakeholders were not able to develop alternative ways to cheat. While cheating practices on paper-based exams continue to be discussed at length in newspaper articles, there have been few reported cases of cheating in the computer-based exams (Biantoro and Arfianti, 2019).^25^ On the other hand, one would expect that the reduction in cheating opportunities would encourage more thorough preparation for exams, which in turn would lead to higher exam scores in schools that switched to CBT. Unfortunately, this learning effect did not yet materialize after three years of implementation.

## CRediT authorship contribution statement

**Emilie Berkhout:** Formal analysis, Methodology, Writing – original draft, Writing – review & editing. **Menno Pradhan:** Conceptualization, Methodology, Writing – original draft, Writing – review & editing. **Rahmawati:** Data curation, Writing – original draft. **Daniel Suryadarma:** Conceptualization, Methodology, Writing – original draft, Writing – review & editing. **Arya Swarnata:** Formal analysis, Writing – original draft.

## Data Availability

Data will be made available on request.

## Appendix A Supplementary tables and figures

MMC S1

## Footnotes

1 In 2010, students had to score higher than 55 out of 100 on average across four subjects to graduate. Between 2011 and 2014, schools gained more autonomy in the graduation of their students when a composite score of the national exam and school exams determined graduation.

2 Although this is true until 2019, admission into higher education levels has not been determined by the national exam since 2020. The 2020 exam was canceled due to the COVID-19 pandemic and the national exam was replaced with a low stakes competency assessment in 2021.

3 From our interview with MoE staff, the qualitative checks did not ultimately change the results. Thus, the index is fully based on the two cheating detection methods.

4 These threshold values are based on the correlation between a change in the integrity index and a change in exam scores between 2015 and 2016. Pusmenjar finds that for schools with an integrity index above 80 in both years that exam scores did not vary much over time, while the exam scores of schools that started with an integrity index below 70 in 2015 and had an integrity index above 80 in 2016 declined substantially (Rahmawati and Asrijanty, 2016).

5 We do not consider later years to check the stability of the integrity index, because CBT seemed to affect the integrity index of surrounding schools through spillover effects (as discussed in Section 6.3).

6 The pairwise correlation coefficient of the integrity index and exam scores in 2015 is −0.6 and is statistically significant at the 1 percent level.

7 Five test versions are shuffled in four different ways to create 20 test booklets per classroom.

8 It is impossible to calculate the integrity index in a comparable way as it is partly based on how often students copy each other’s answers. In CBT, every student receives different questions.

9 A CD with the national examination data, including the integrity index, can be requested from the Ministry of Education. Exam score data between 2015 and 2019, but not the integrity index, can also be accessed at https://hasilun.pusmenjar.kemdikbud.go.id/.

10 We perform the same exercise on the full sample and find similar but less distinct patterns, see Table A2 in Appendix.

11 The results are similar if we add not-yet-treated schools in the comparison group (Figure A1). We also implement further robustness checks in Section 6.4.

12 Since the exam has not been a graduation requirement since 2015, we do not analyze passing rates.

13 Note that any time-invariant differences between districts are also taken up by these school fixed effects. Fig. 2 suggests that this may be relevant as cheating was regionally clustered.

14 In Figure A2, we show that results are similar when we estimate the model on the unbalanced panel, that is, when we drop the restriction of complete exam data for all years.

15 While statistically insignificant, the point estimates are negative. Under the assumption that those most affected by CBT (cheaters) would switch schools, our estimates provide a lower bound for the drop in test scores.

16 This analysis includes 30,198 out of 50,124 schools for which both the integrity score and school resource information are available.

17 Figure A5 also shows impact estimates on the integrity index after CBT implementation. Note that these estimates are mechanical, as the integrity index is assumed to be 100 when schools implement CBT.

18 We do not have access to the within school standard deviation of exam scores in 2019, so for that year we assume that the ratio between the sum of squares across groups and the sum of squares within groups is the same as in 2018.

19 We only show first year average effects as this is the only period for which all cohorts contribute to the estimated effect.

20 We find a similar pattern in differences in average scores by subject among schools with an integrity index above 70 before they switched to CBT in 2015: 67.2 in Indonesian, 52.7 in English, 52.8 in Science and 48.6 in Mathematics. This also suggests that students found mathematics the most difficult subject to achieve a high score on, and Indonesian the easiest.

21 One might be concerned that the impacts on these four groups are not directly comparable due to correlations between cheating and computer availability: low integrity schools are less likely to have a computer lab in 2015 (see Table A1). The integrity index of low integrity schools with a computer lab is 3.4 points higher than the integrity index of low integrity schools without a computer lab (not shown in the table). This means that the difference in impacts between those two groups is not only explained by lack of computer skills but also by more initial cheating. Hence, the impacts from computer familiarity are likely to be even smaller than shown here.

22 Evidence for limited effects from the test taking mode were also found in the US (Wang et al., 2008) and India (Singh, 2020), where computer-based testing yielded similar results as compared to paper-based testing when there was no scope for cheating in either.

23 Note that there might still be cheating in schools with integrity above 70, as described in Section 2.

24 The part of the drop in exam scores explained by lack of computer availability is 0.8 points (6.6–5.8), while part of the drop explained by low integrity is 3.2 points (6.6–3.4).

25 There was one teacher that managed to connect his computer with those of the students such that he could control their computers from a distance (Abdi, 2019) and there were some students who took photos of the computer screen during the exam to share questions with others (Alfons, 2019).
