# Supplementary material for: Using technology to prevent fraud in high stakes national school examinations: Evidence from Indonesia
Source: J Dev Econ. 2024 Sep;170:103307. doi: 10.1016/j.jdeveco.2024.103307 (PMC11216028; doi:10.1016/j.jdeveco.2024.103307)
Supplement: MMC S1 [file mmc1.pdf]

A Appendix - Tables

Table A1: Difference Between Schools with an Integrity Score Above and Below 70

2015 Variables	(1) Integrity < 70	(2) Integrity >= 70	(3) Difference
Exam Score	71.28	55.53	-15.75*
	(9.42)	(10.83)	[0.66]
Number of Exam Participants	77.19	105.97	28.77*
	(76.34)	(94.42)	[2.63]
Student-Teacher Ratio[1]	13.92	15.63	1.71*
	(7.22)	(7.94)	[0.33]
Share of teachers with 4-year degree[1]	0.84	0.87	0.03*
	(0.18)	(0.16)	[0.01]
Share of teachers that are civil servant[1]	0.50	0.46	-0.03
	(0.34)	(0.37)	[0.02]
Public School	0.41	0.43	0.02
	(0.49)	(0.49)	[0.02]
Rural[1]	0.75	0.68	-0.07*
	(0.44)	(0.47)	[0.04]
Electricity[1]	0.94	0.99	0.05*
	(0.24)	(0.10)	[0.01]
Internet[1]	0.83	0.89	0.06*
	(0.37)	(0.31)	[0.01]
Computer Lab[1]	0.39	0.56	0.17*
	(0.49)	(0.50)	[0.02]
Observations	16,439	27,747	50,124

Notes: [1] Number of observations are lower for these variables: 19,917 schools with an integrity index above 70 and 10,262 schools with an integrity index below 70. The number of observations for the availability of a computer lab are 20,473 schools with an integrity index above 70 and 10,419 schools with an integrity index below 70. The table includes panel schools that participated in the exam each year between 2015 and 2019. Standard deviations between parentheses and standard errors between brackets, corrected for clustering that the district level. * p<0.05

Table A2: Rank Correlation over Time for All Districts

	(1)<br>School Percentile	(2)<br>School Rank Within District	(3)<br>District Rank
2011	0.37	0.63	0.42
2012	0.43	0.64	0.50
2013	0.54	0.73	0.60
2014	0.62	0.77	0.69
2015	1	1	1
2016	0.65	0.81	0.71
2017	0.50	0.76	0.61
2018	0.31	0.69	0.32
2019	0.24	0.68	0.21
Observations	50,084	50,084	514

Note: Table shows the Pearson pairwise correlation coefficient of the rank in each year with the rank in 2015. It includes 50,084 panel schools from 514 districts, only excluding 40 schools that switched to CBT in 2015. None of the schools in the table implemented CBT in 2015, 2 percent in 2016, 20 percent in 2017, 53 percent in 2018 and 79 percent in 2019. There are between 6 and 952 schools in a district (107 on average).

Table A3: Impact Estimation Result for Raw Exam Scores

Dependent Variable: Exam Score	(1) 2017 Cohort	(2) 2018 Cohort	(3) 2019 Cohort	(4) Combined
CBT -9			1.64	
			(1.12)	
CBT -8		0.73	2.04	
		(1.10)	(1.09)	
CBT -7	-0.80	1.00	1.15	0.57
	(1.20)	(1.21)	(1.15)	(1.02)
CBT -6	-2.52	0.93	-0.10	-0.32
	(1.26)	(1.23)	(1.00)	(0.98)
CBT -5	-1.91	0.23	-0.99	-0.73
	(1.19)	(1.00)	(0.98)	(0.82)
CBT -4	-0.51	-1.45	0.38	-0.61
	(0.87)	(0.96)	(0.63)	(0.65)
CBT -3	-3.18	0.21	0.50	-0.60
	(0.84)*	(0.64)	(0.38)	(0.38)
CBT -2	0.00	0.00	0.00	0.00
CBT -1	-1.24	-1.85	-1.91	-1.70
	(0.79)	(0.43)*	(0.49)*	(0.30)*
CBT 0	-5.21	-7.45	-5.69	-6.30
	(0.86)*	(0.82)*	(0.69)*	(0.56)*
CBT 1	-2.52	-6.78		
	(1.02)	(0.75)*		
CBT 2	-1.67			
	(1.01)			
School Fixed Effects	Yes	Yes	Yes	Yes
Year Fixed Effects	Yes	Yes	Yes	Yes
Number of Schools	15,468	20,151	17,102	39,420
Comparison Mean t=0	52.30	49.99	50.80	

Note: Standard errors between parentheses and corrected for clustering that the district level. The “combined” columns show the sample-weighted average effect across cohorts. * p<0.05

Table A4: Impact Estimation Result for Standardized Exam Scores

Dependent Variable: Standardized Exam Score	(1) 2017 Cohort	(2) 2018 Cohort	(3) 2019 Cohort	(4) Combined
CBT -8			0.20	
			(0.010)	
CBT -7		0.17	0.10	0.14
		(0.10)	(0.09)	(0.10)
CBT -6	-0.16	0.13	-0.01	0.01
	(0.10)	(0.09)	(0.08)	(0.08)
CBT -5	-0.11	0.05	-0.08	-0.04
	(0.09)	(0.08)	(0.08)	(0.05)
CBT -4	-0.01	-0.10	0.02	-0.04
	(0.07)	(0.07)	(0.05)	(0.05)
CBT -3	-0.23	0.02	0.03	-0.05
	(0.06)*	(0.05)	(0.03)	(0.03)
CBT -2	0.00	0.00	0.00	0.00
CBT -1	-0.09	-0.12*	-0.15	-0.12
	(0.06)	(0.04)	(0.04)*	(0.02)*
CBT 0	-0.39	-0.56	-0.45	-0.48
	(0.06)*	(0.06)*	(0.05)*	(0.04)*
CBT 1	-0.18	-0.52		
	(0.08)	(0.06)*		
CBT 2	-0.11			
	(0.07)			
School Fixed Effects	Yes	Yes	Yes	Yes
Year Fixed Effects	Yes	Yes	Yes	Yes
Number of Schools	15,468	20,151	17,102	39,420
Comparison Mean t=0	-0.03	-0.03	-0.03	

Note: Standard errors between parentheses and corrected for clustering that the district level. Outcome is standardized using the student-level comparison group mean and standard deviation in each year. The “combined” columns show the sample-weighted average effect across cohorts. * p<0.05

Table A5: Impact Estimation Result for Raw Exam Scores by Subject

Dependent Variable: Exam Score	(1) Indonesian	(2) English	(3) Science	(4) Mathematics
CBT -7	-0.12	0.37	0.87	1.14
	(0.65)	(1.19)	(1.15)	(1.50)
CBT -6	-0.31	-0.62	0.02	-0.36
	(0.61)	(1.24)	(1.10)	(1.25)
CBT -5	-0.88	-1.10	-0.50	-0.45
	(0.55)	(1.05)	(0.97)	(1.04)
CBT -4	-0.76	-0.43	-0.62	-0.66
	(0.41)	(0.76)	(0.71)	(0.85)
CBT -3	-0.66	-0.37	-0.70	-0.67
	(0.24)*	(0.43)	(0.44)	(0.46)
CBT -2	0.00	0.00	0.00	0.00
CBT -1	-0.91	-1.87	-1.73	-2.31
	(0.24)*	(0.35)*	(0.36)*	(0.40)*
CBT 0	-3.32	-6.70	-5.76	-9.40
	(0.40)*	(0.59)*	(0.62)*	(0.80)*
School Fixed Effects	Yes	Yes	Yes	Yes
Year Fixed Effects	Yes	Yes	Yes	Yes
Number of Schools	39,420	39,420	39,420	39,420
Treatment Mean t=0	60.9	44.7	44.6	40.1

Note: We show mean values of the treatment group here to indicate that most cheating happened in subjects that students struggled with most. Therefore, we show scores that do not include cheating. Standard errors between parentheses and corrected for clustering that the district level. Table shows the sample-weighted average effect across cohorts. * p<0.05

Table A6: Heterogeneous Impact Estimation Result for Raw Exam Scores

Dependent Variable: Exam Score	(1)	(2)	(3)	(4)	(5)	(6)	(7)	(8)	(9)	(10)	(11)	(12)
	Integrity >= 70						Integrity < 70					
	No Computers			Computers			No Computers			Computers		
	2017	2018	2019	2017	2018	2019	2017	2018	2019	2017	2018	2019
CBT -9			1.54			-0.06			3.77			1.94
			(0.91)			(1.61)			(1.11)*			(1.83)
CBT -8		1.10	0.91		-0.62	-0.27		1.00	4.07		-0.18	1.45
		(1.12)	(1.09)		(1.55)	(1.67)		(1.18)	(1.26)*		(1.64)	(2.02)
CBT -7	-2.44	0.43	1.29	-8.79	-1.41	-1.24	0.69	1.85	4.39	0.63	-0.05	1.65
	(1.16)	(1.34)	(1.02)	(1.42)*	(1.65)	(2.09)	(1.98)	(1.15)	(1.14)*	(1.40)	(1.88)	(2.05)
CBT -6	-4.25	0.47	0.23	-9.96	-1.43	-0.27	1.57	1.62	2.42	0.11	0.14	-0.11
	(1.38)*	(1.00)	(0.98)	(1.55)*	(1.89)	(1.69)	(2.28)	(1.14)	(1.16)	(1.75)	(1.88)	(1.88)
CBT -5	-3.26	0.01	-0.33	-8.25	0.10	-0.45	1.32	-0.81	1.94	0.68	-1.01	0.27
	(1.34)	(1.33)	(0.99)	(1.72)*	(1.73)	(1.61)	(2.13)	(1.38)	(1.13)	(1.59)	(1.87)	(1.98)
CBT -4	-2.73	-0.91	0.61	-3.01	-0.73	1.21	-2.03	-2.39	4.03	-1.52	-3.42	2.09
	(0.99)	(1.28)	(0.65)	(1.18)	(1.43)	(0.99)	(2.09)	(1.25)	(0.96)*	(1.58)	(1.89)	(1.60)
CBT -3	-3.79	0.07	-0.53	-4.81	1.77	-0.87	-4.16	2.13	0.85	-4.27	0.26	1.13
	(0.98)*	(0.80)	(0.55)	(1.14)*	(1.08)	(0.68)	(1.82)	(0.98)	(0.60)	(1.36)*	(1.53)	(1.13)
CBT -2	0.00	0.00	0.00	0.00	0.00	0.00	0.00	0.00	0.00	0.00	0.00	0.00
CBT -1	-1.48	-0.02	-0.72	-4.40	0.18	-0.88	-5.96	-3.21	-3.33	-1.76	-3.73	-2.75
	(0.95)	(0.67)	(0.52)	(0.98)*	(0.73)	(0.83)	(2.28)	(0.69)*	(0.79)*	(1.64)	(1.05)*	(1.51)
CBT 0	-4.05	-2.78	-2.27	-6.78	-1.89	-2.07	-17.69	-14.65	-8.99	-12.30	-9.79	-6.84
	(0.91)*	(1.03)	(0.71)*	(0.99)*	(1.14)	(1.00)	(2.02)*	(1.11)*	(0.94)*	(1.73)*	(1.88)*	(1.45)*
CBT 1	-1.48	-2.82		-4.43	-1.43		-14.97	-13.48		-7.88	-8.62	
	(1.00)	(1.00)		(1.04)*	(1.10)		(2.38)*	(1.08)*		(2.16)*	(1.72)*	
CBT 2	-1.33			-3.57			-13.45			-6.83		
	(0.96)			(1.08)*			(2.42)*			(1.90)*		
School Fixed Effects	Yes	Yes	Yes	Yes	Yes	Yes	Yes	Yes	Yes	Yes	Yes	Yes
Year Fixed Effects	Yes	Yes	Yes	Yes	Yes	Yes	Yes	Yes	Yes	Yes	Yes	Yes
Number of Schools	3,777	4,082	4,348	4,889	3,549	2,705	2,429	3,221	3,310	1,248	1,458	1,208
Comparison Mean t=0	48.71	46.54	48.00	50.43	48.62	49.47	56.21	54.31	54.78	56.21	51.93	52.44

Note: Table only includes schools for which the integrity index and computer information is available in 2015. Standard errors between parentheses and corrected for clustering that the district level. Table shows the sample-weighted average effect across cohorts. * p<0.05

Table A7: Impact Estimation Result for Standard Deviation of Exam Scores within Schools

Dependent Variable: S.D Within School	(1) 2017 Cohort	(2) 2018 Cohort	(3) Combined
CBT -8		-0.64	
		(0.09)*	
CBT -7	-0.83	-0.42	-0.58
	(0.13)*	(0.09)*	(0.09)*
CBT -6	-0.16	-0.37	-0.29
	(0.13)	(0.09)*	(0.08)*
CBT -5	-0.35	0.13	-0.06
	(0.13)*	(0.07)	(0.07)
CBT -4	-0.21	0.15	0.01
	(0.11)	(0.07)	(0.06)
CBT -3	-0.03	-0.07	-0.05
	(0.09)	(0.05)	(0.04)
CBT -2	0.00	0.00	0.00
CBT -1	0.29	-0.08	0.07
	(0.09)*	(0.06)	(0.05)
CBT 0	0.77	0.52	0.62
	(0.12)*	(0.08)*	(0.07)*
CBT 1	0.29		
	(0.09)		
School Fixed Effects	Yes	Yes	Yes
Year Fixed Effects	Yes	Yes	Yes
Number of Schools	15,468	20,151	35,619
Comparison Mean t=0	5.34	5.84	

Note: Standard errors between parentheses and corrected for clustering that the district level. Table shows the sample-weighted average effect across cohorts. * $p<0.05$

B Appendix - Figures

Figure A1: Impact Results Using Not Yet Treated Schools as Comparison Group

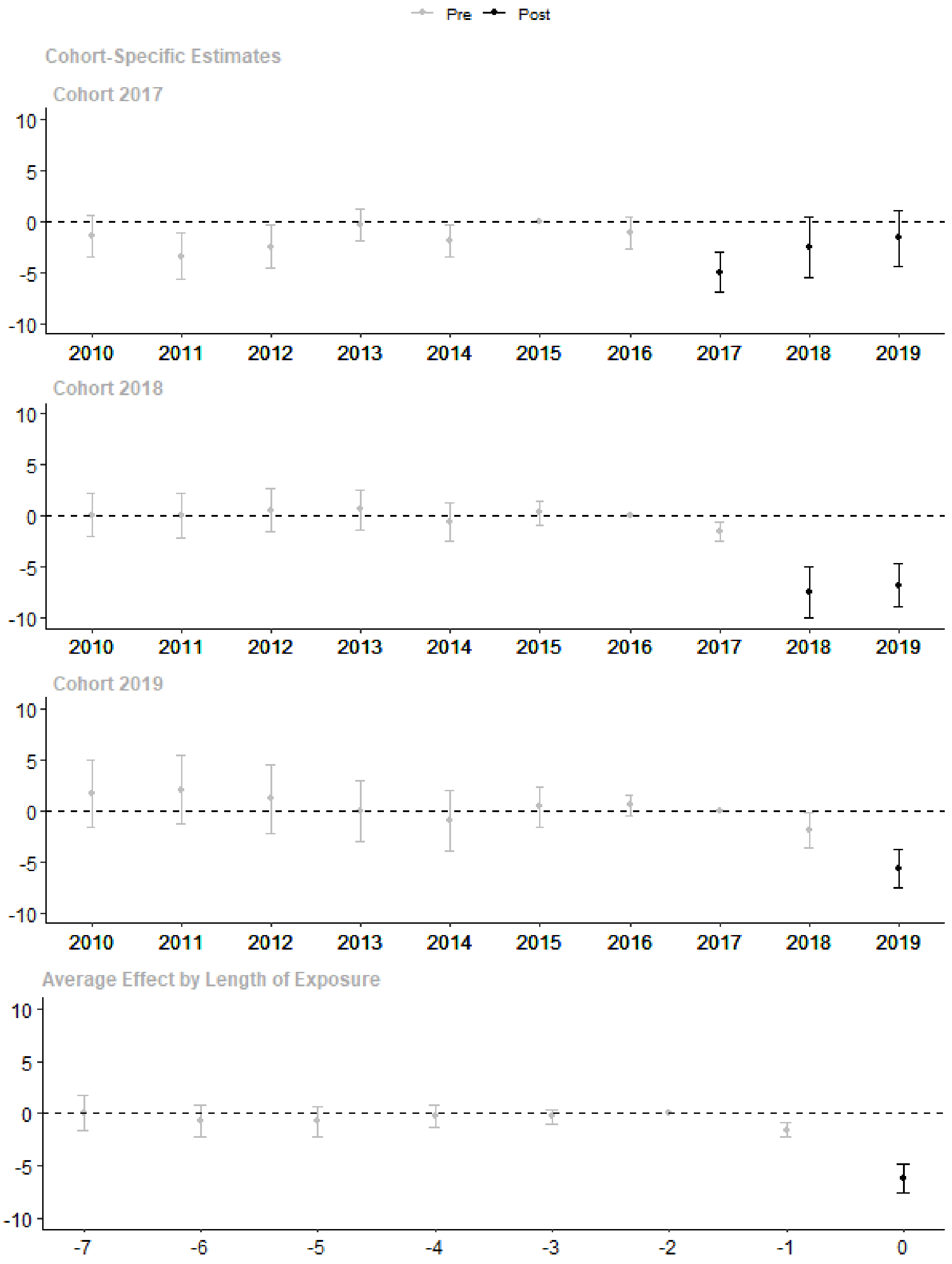

Note: Plot of post-CBT point estimates of β_e and pre-CBT point estimates of δ_e in equation 1 with 95% confidence interval, estimated separately for each cohort. Standard errors are corrected for clustering at the district level. The “average effect by length of exposure” figure shows the sample-weighted average effect across cohorts. Never-treated schools are always part of the control group. The not-yet-treated schools are added in relevant cohorts and years.

Figure A2: Impact Results Using Unbalanced Panel Schools

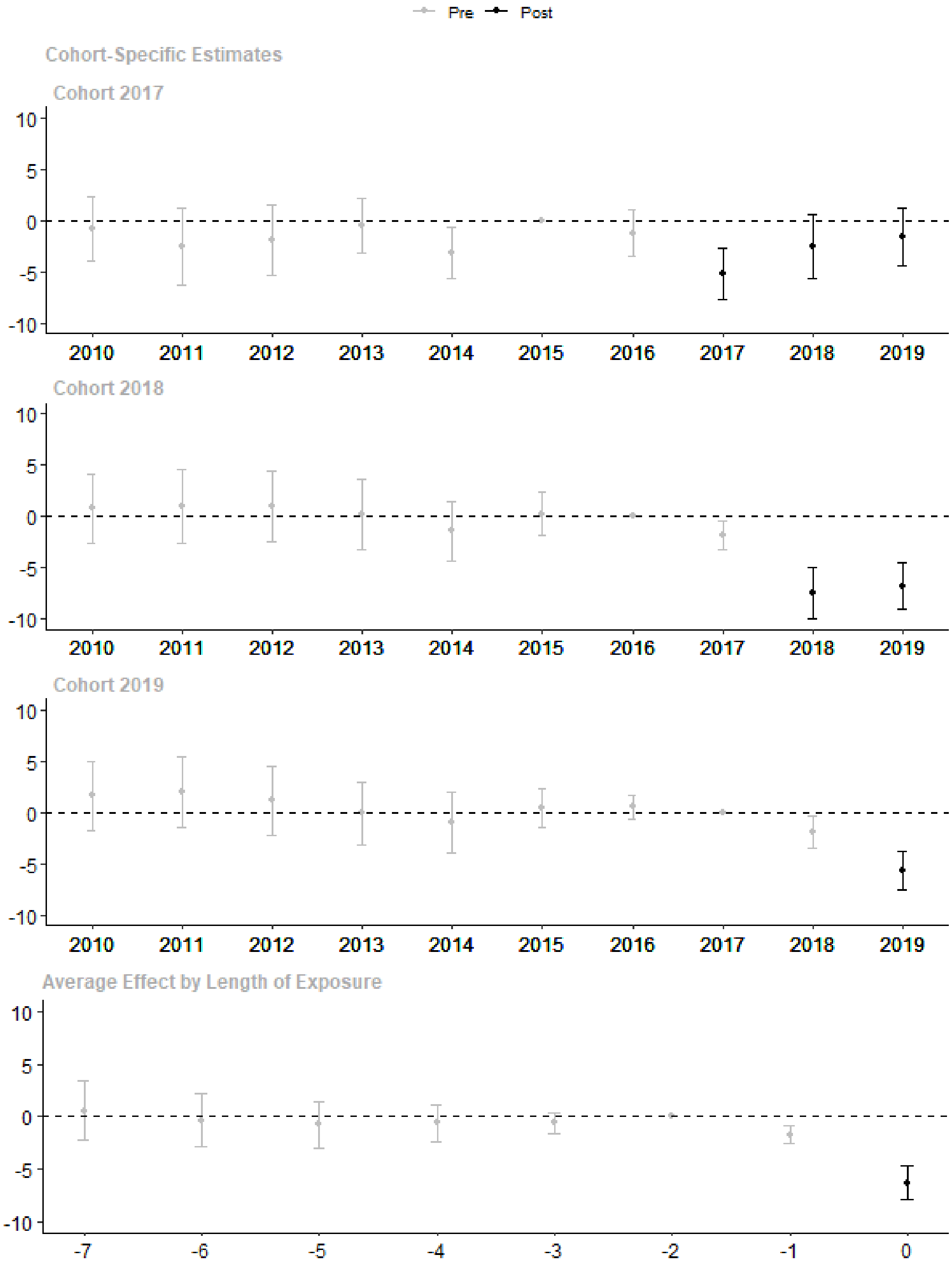

Note: Plot of post-CBT point estimates of β_e and pre-CBT point estimates of δ_e in equation 1 with 95% confidence interval, estimated separately for each cohort. Standard errors are corrected for clustering at the district level. The "average effect by length of exposure" figure shows the sample-weighted average effect across cohorts.

Figure A3: Common Support Between comparison schools and Each Treatment Cohort

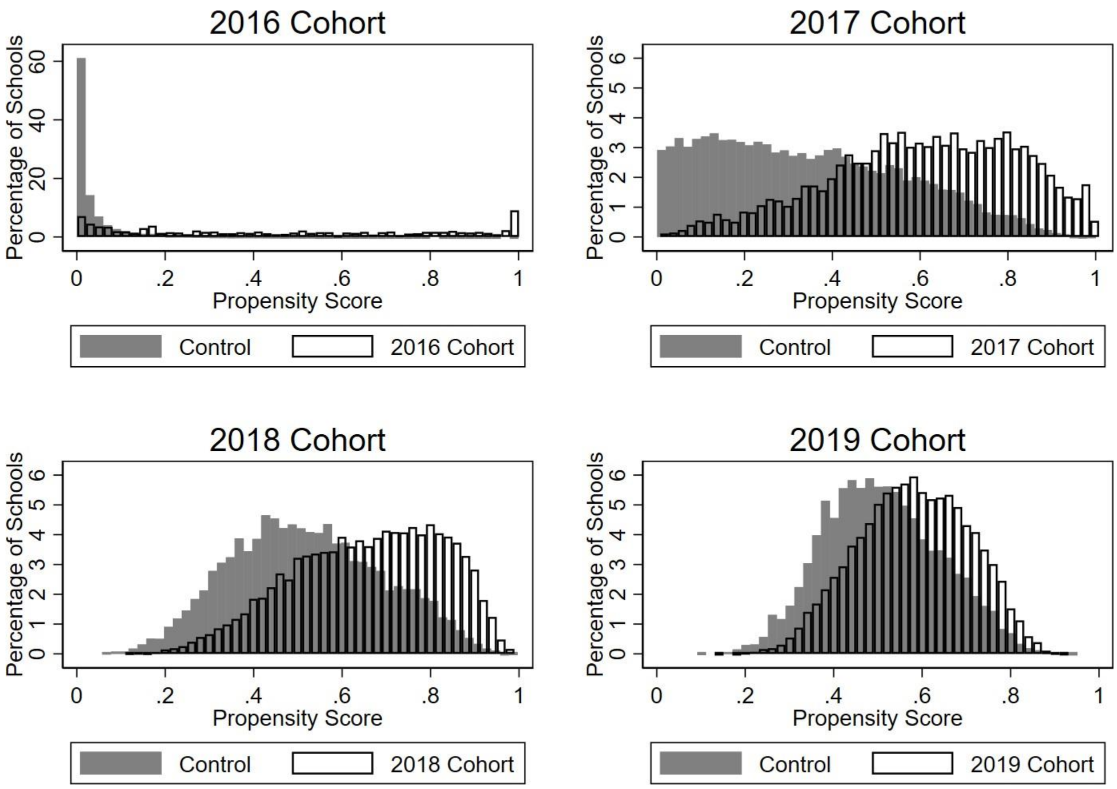

Note: Propensity score is estimated using the school average exam score in each year from 2010 to 2015 and the integrity index in 2015. Size of bins is 0.02. Y-axis scale of the first histogram that compares the comparison schools to the schools that switched to CBT in 2016 deviates from the scale of the other figures.

Figure A4: Impact Results on Exam Participants

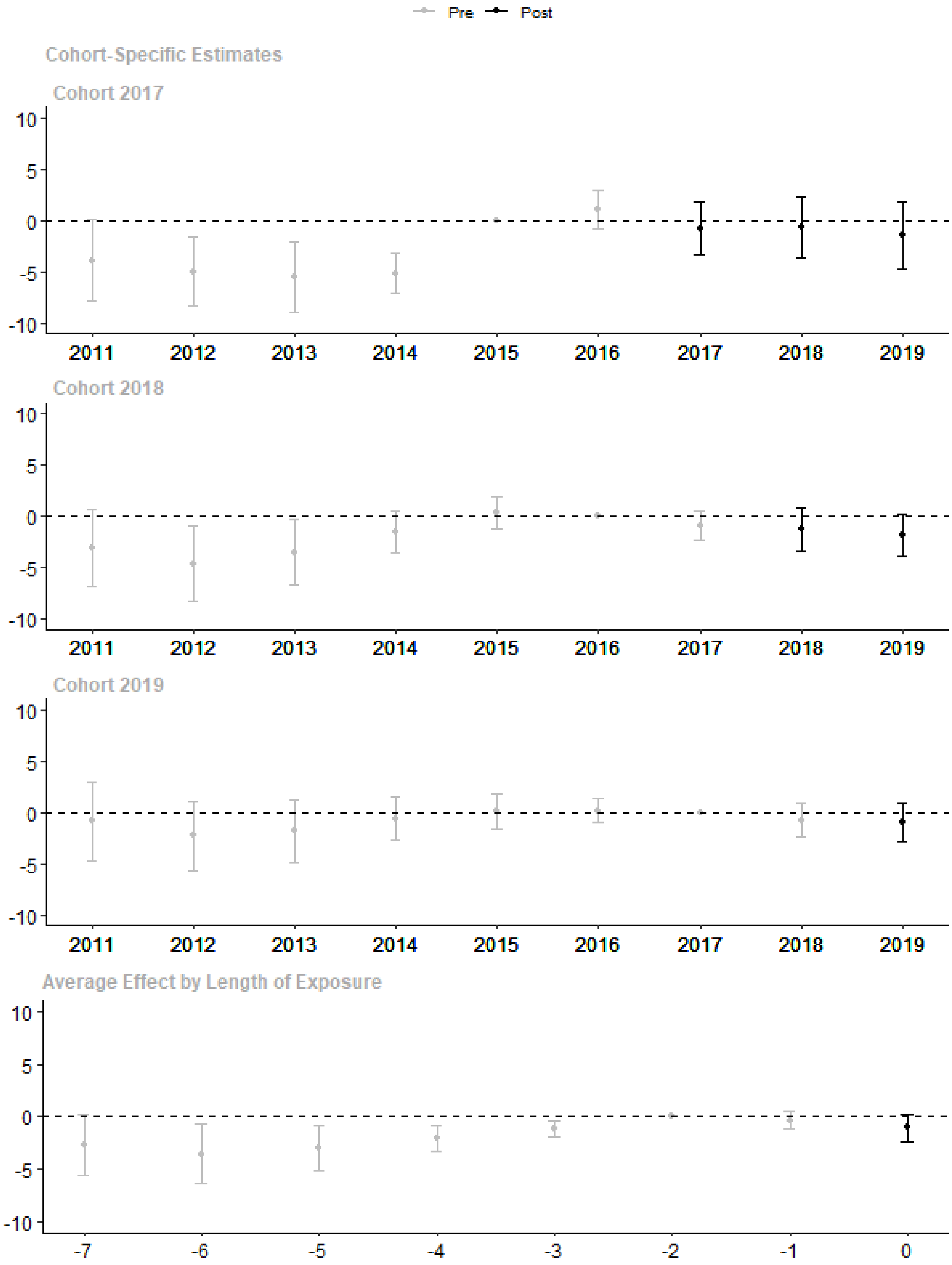

Note: Plot of post-CBT point estimates of β_e and pre-CBT point estimates of δ_e in equation 1 with 95% confidence interval, estimated separately for each cohort. Standard errors are corrected for clustering at the district level. The "average effect by length of exposure" figure shows the sample-weighted average effect across cohorts.

Figure A5: Impact Estimation Result on Integrity Index

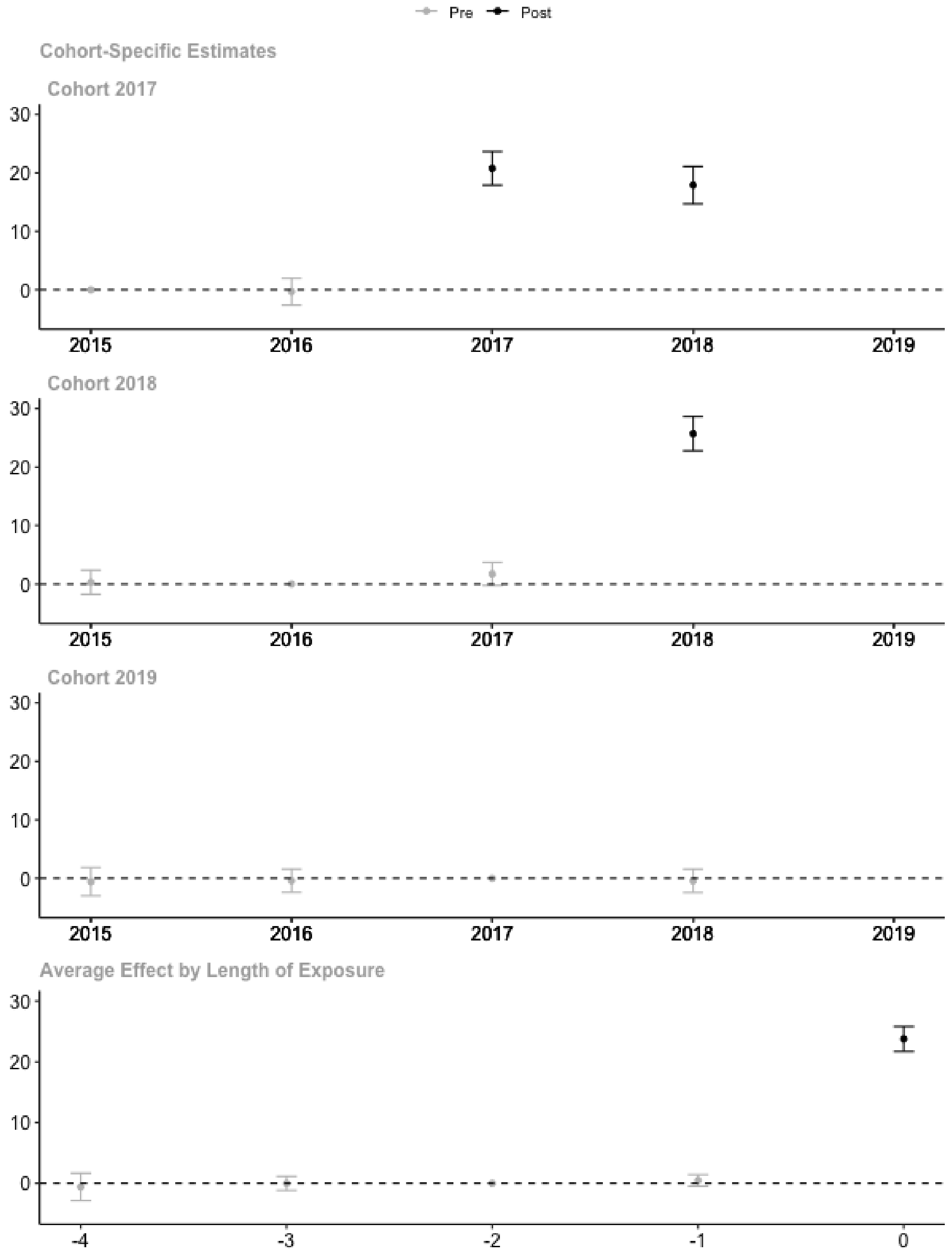

Note: Plot of post-CBT point estimates of β_e and pre-CBT point estimates of δ_e in equation 1 with 95% confidence interval, estimated separately for each cohort. Estimates are missing in 2019 because the integrity index is not available in that year. Impact estimates in the post-periods are mechanical, since the integrity index is assumed to be 100 when schools implement CBT. Standard errors are corrected for clustering at the district level. The "average effect by length of exposure" figure shows the sample-weighted average effect across cohorts.

Figure A6: Impact Results on Exam Scores by Integrity Index Category

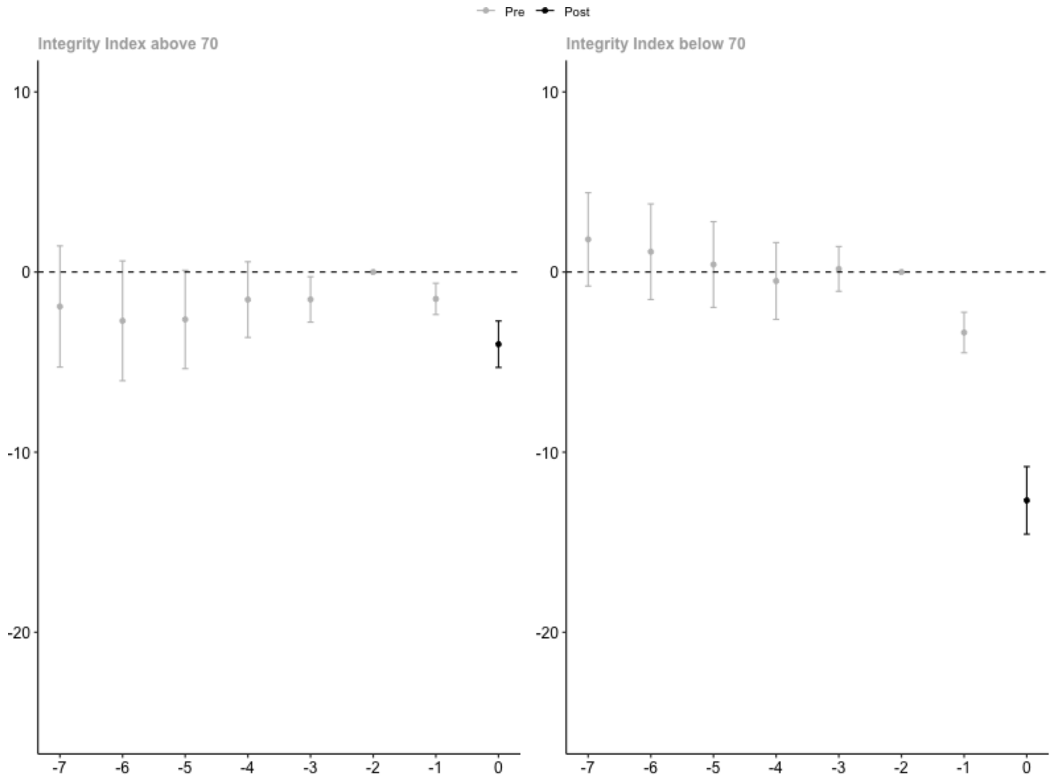

Note: Plot of post-CBT point estimates of β_e and pre-CBT point estimates of δ_e in equation 1 with 95% confidence interval, estimated separately for each integrity category. The figure includes 44,226 schools for which the integrity index is available in 2015. The integrity categories are based on the integrity index in 2015. Standard errors are corrected for clustering at the district level.

Figure A7: Within-School Score Variance Distribution by Integrity and Over Time

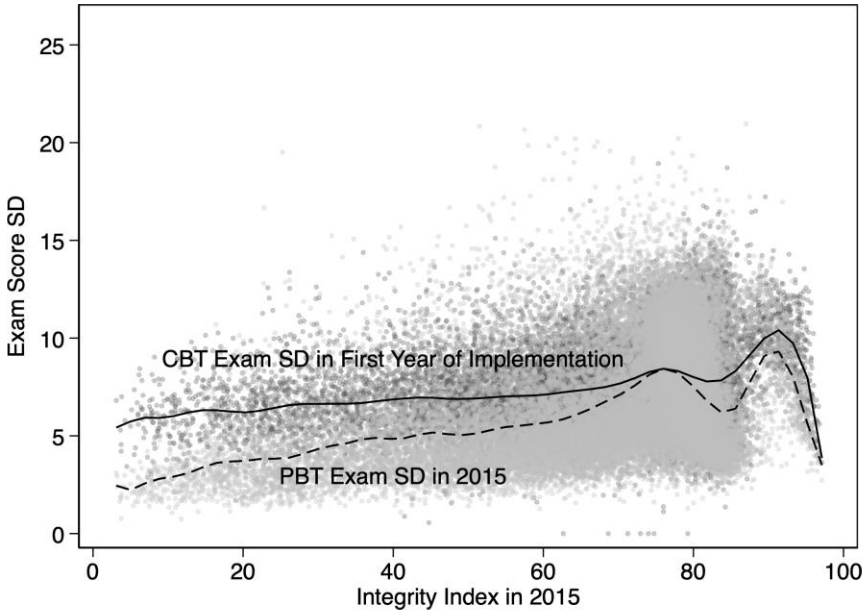

(a) Difference between 2015 PBT and first CBT Within-School Score Variance by Integrity

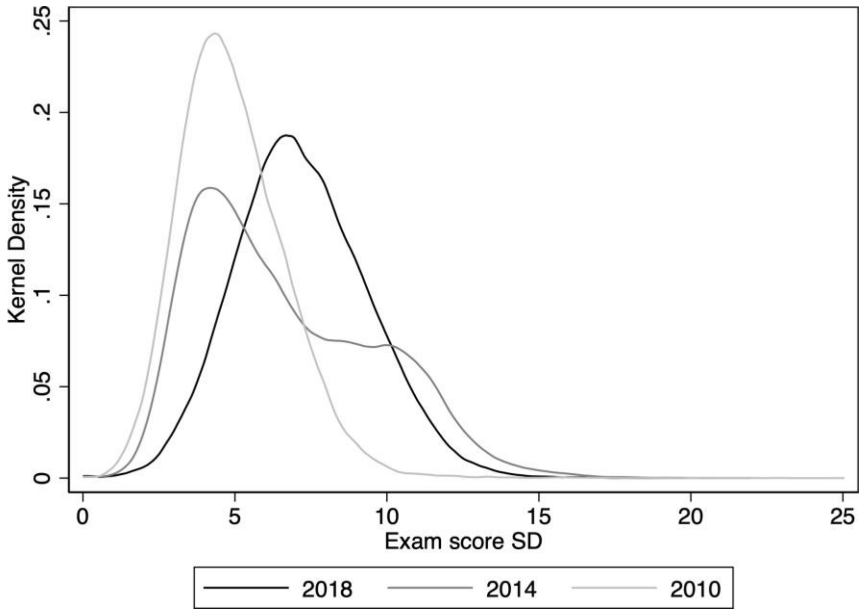

(b) Change in Within-School Exam Score Variance Distribution between 2010 and 2018

Note: The figure includes 34,783 out of 39,420 treatment schools for which the 2015 integrity index is non-missing. The CBT score polynomial regression result combines the exam scores of all treatment schools in the first year of CBT implementation, which is between 2016 and 2019. The within-school standard deviation of the exam scores is not available in 2019.

Figure A8: Conditional Impact Results

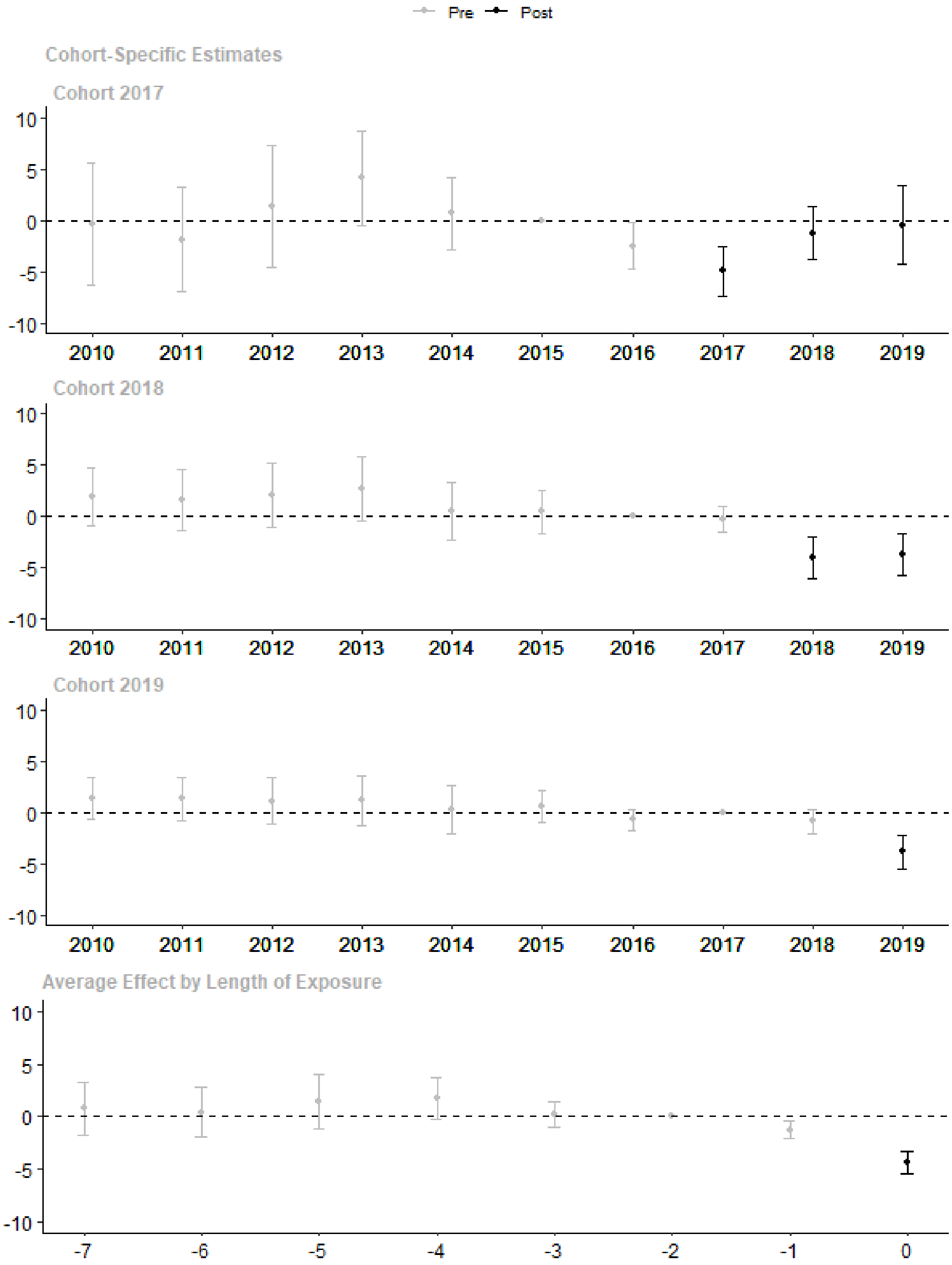

Note: Results conditional on the following school characteristics in 2015: exam score, integrity index, number of students, student-teacher ratio, public school, share of teachers with 4-year degree, rural, electricity, internet, computers and island group. Plot of post-CBT point estimates of β_e and pre-CBT point estimates of δ_e in equation 1 with 95% confidence interval, estimated separately for each cohort. Standard errors are corrected for clustering at the district level. The "average effect by length of exposure" figure shows the sample-weighted average effect across cohorts.

Figure A9: Impact Results Conditional on Pre-trend Between 2010 and 2015

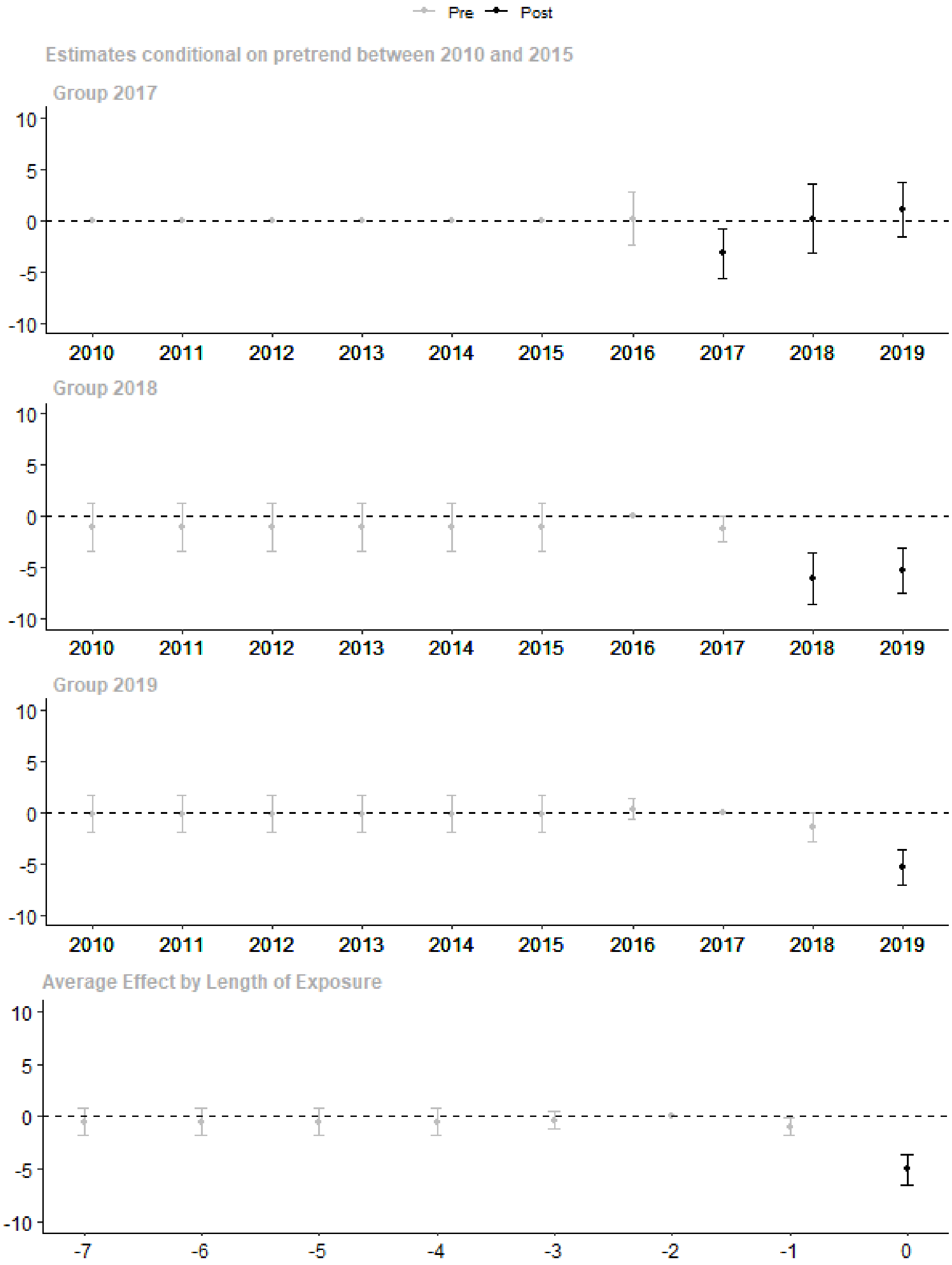

Note: Results conditional on the exam scores in each year between 2010 and 2015. Matching makes the treatment and comparison group perfectly similar in terms of their exam scores from 2010 to 2015. Because 2015 is the base year for the 2017 cohort, there are no estimates for the pretrend for that cohort. The pretrend estimates for the 2018 and 2019 show the difference between the pretrend and the base years 2016 and 2017, respectively, so the results are the same across the pretrend years for those cohorts. Plot of post-CBT point estimates of β_e and pre-CBT point estimates of δ_e in equation 1 with 95% confidence interval, estimated separately for each cohort. Standard errors are corrected for clustering at the district level. The "average effect by length of exposure" figure shows the sample-weighted average effect across cohorts.
